# A metabolic checkpoint protein GlmR is important for diverting carbon into peptidoglycan biosynthesis in *Bacillus subtilis*

**DOI:** 10.1371/journal.pgen.1007689

**Published:** 2018-09-24

**Authors:** Vaidehi Patel, Qun Wu, Pete Chandrangsu, John D. Helmann

**Affiliations:** 1 Cornell University, Department of Microbiology, Ithaca, NY, United States of America; 2 School of Biotechnology, Jiangnan University, Wuxi, China; Indiana University, UNITED STATES

## Abstract

The *Bacillus subtilis* GlmR (formerly YvcK) protein is essential for growth on gluconeogenic carbon sources. Mutants lacking GlmR display a variety of phenotypes suggestive of impaired cell wall synthesis including antibiotic sensitivity, aberrant cell morphology and lysis. To define the role of GlmR, we selected suppressor mutations that ameliorate the sensitivity of a *glmR* null mutant to the beta-lactam antibiotic cefuroxime or restore growth on gluconeogenic carbon sources. Several of the resulting suppressors increase the expression of the GlmS and GlmM proteins that catalyze the first two committed steps in the diversion of carbon from central carbon metabolism into peptidoglycan biosynthesis. Chemical complementation studies indicate that the absence of GlmR can be overcome by provision of cells with N-acetylglucosamine (GlcNAc), even under conditions where GlcNAc cannot re-enter central metabolism and serve as a carbon source for growth. Our results indicate that GlmR facilitates the diversion of carbon from the central metabolite fructose-6-phosphate, which is limiting in cells growing on gluconeogenic carbon sources, into peptidoglycan biosynthesis. Our data suggest that GlmR stimulates GlmS activity, and we propose that this activation is antagonized by the known GlmR ligand and peptidoglycan intermediate UDP-GlcNAc. Thus, GlmR presides over a new mechanism for the regulation of carbon partitioning between central metabolism and peptidoglycan biosynthesis.

## Author summary

Bacterial cells are surrounded by a peptidoglycan cell wall that is, under most conditions, required for viability. Synthesis of the cell wall requires a considerable diversion of resources from central carbon metabolism into a lipid-linked precursor (lipid II) that is exported from the cell for wall assembly. Here, we propose that GlmR presides over a new mechanism for the regulation of carbon partitioning between central metabolism and peptidoglycan biosynthesis: GlmR activates the GlmS-dependent diversion of carbon from the glycolytic pathway into peptidoglycan synthesis. This effect is particularly important during gluconeogenesis since the GlmS substrate fructose 6-phosphate is present at a reduced level under these conditions.

## Introduction

*Bacillus subtilis* provides a powerful model system for understanding cell wall homeostasis in Gram positive bacteria. Disruption of pathways for the synthesis of peptidoglycan (PG) and other cell envelope components elicits complex adaptive responses often controlled by alternative σ factors or two-component systems [1, 2]. The ECF σ factor σ^M^ regulates numerous operons involved in PG synthesis and mutants are sensitive to PG synthesis inhibitors [3]. Previously, we found that mutation of *gdpP*, which encodes a cyclic-di-adenosine monophosphate (c-di-AMP) hydrolase, can suppress the sensitivity of *B*. *subtilis sigM* null mutants towards beta-lactam antibiotics [4]. This suggests that c-di-AMP plays some role in PG homeostasis. Mutations in the *yvcK* gene (herein renamed *glmR*) also exhibit cell envelope defects, as evidenced by cell bulging and lysis when inoculated into non-glycolytic carbon sources [5]. Moreover, a *yqfF*::*Tn* insertion suppressed the inability of a *glmR* mutant to grow on gluconeogenic media [5]. Although unknown at the time, *yqfF* is now known to encode a second c-di-AMP hydrolase renamed PgpH [6, 7]. These observations encouraged us to investigate possible connections between GlmR, c-di-AMP, and cell envelope homeostasis.

In *B*. *subtilis*, GlmR (formerly YvcK) is essential for growth on amino acids and intermediates of the tricarboxylic acid cycle and pentose phosphate pathway, but dispensable for growth on glucose and other glycolytic carbon sources [5]. Previous genetic studies revealed that mutations in genes affecting central carbon metabolism (CCM), including *zwf* and *cggR*, allow a *glmR* null mutant to grow on gluconeogenic carbon sources [5]. These observations suggest that GlmR has a yet undefined role in regulating metabolism. In the absence of GlmR, cells display cell envelope defects and lyse under gluconeogenic growth conditions.

The function of GlmR in CCM, and how this relates to cell envelope integrity, is not yet clear. One model suggests that GlmR may function as a cytoskeletal filament protein analogous to MreB to help coordinate cell wall synthesis [8]. MreB, an actin-like cytoskeletal protein, is important for maintaining a rod shape in *B*. *subtilis* and deletion of *mreB* leads to severe morphological defects and eventual cell lysis, effects attributed to mislocalization of penicillin binding protein 1 (PBP1) [9]. *B*. *subtilis* GlmR localizes to the membrane in a helical fashion, and overexpression of GlmR rescues the cell defects seen in an *mreB* deletion mutant and restores proper localization of PBP1. Conversely, overexpression of MreB rescues the morphological defects of a *glmR* null mutant when grown on gluconeogenic carbon sources [8].

Recently, GlmR was found to possess a ligand binding site for UDP sugars such as UDP-glucose and UDP-N-acetylglucosamine (UDP-GlcNAc) [10]. Since UDP-GlcNAc is a precursor of PG synthesis, this suggests that GlmR may sense this intermediate to somehow modulate CCM or cell envelope homeostasis. Mutations altering the UDP-sugar binding site did not affect growth on gluconeogenic media in *B*. *subtilis*, but did lead to increased sensitivity to bacitracin [10].

Although the biochemical details are unclear, the role of GlmR in metabolism and cell wall homeostasis seems to be widely conserved. Homologs of GlmR are present diverse bacteria and a *glmR* mutant can be complemented by expression of the *Escherichia coli* homolog, YbhK [5]. Mutation of *glmR* homologs in the intracellular pathogens *Mycobacterium tuberculosis* (*cuvA*) and *Listeria monocytogenes* (*yvcK*) leads to alterations in cell morphology and sensitivity to cell wall acting antibiotics, as well as defects in carbon source utilization and establishment of infection in the host cell [11, 12]. Although these diverse phenotypes, biochemical properties and cell localization studies are all intriguing, a unifying model to account for the role of GlmR in the cell has been elusive.

Here, we show that a *B*. *subtilis* strain lacking *glmR* is susceptible to peptidoglycan (PG) biosynthesis inhibitors such as beta-lactams, vancomycin and moenomycin. Characterization of *glmR* suppressor mutations indicates that increased expression of genes involved in UDP-GlcNAc biosynthesis is sufficient to increase beta-lactam resistance and restore growth on gluconeogenic carbon sources. Moreover, supplementation with GlcNAc can bypass the requirement for GlmR even in strains where GlcNAc cannot enter into CCM. Our results support a model in which GlmR functions to help divert carbon to PG biosynthesis, likely through direct interaction with GlmS. We propose that this effect is particularly important during gluconeogenesis since the GlmS substrate fructose 6-phosphate is present at a reduced level under these conditions [13].

## Results

### Δ*glmR* is sensitive to peptidoglycan synthesis inhibiting antibiotics

**Fig 1:**
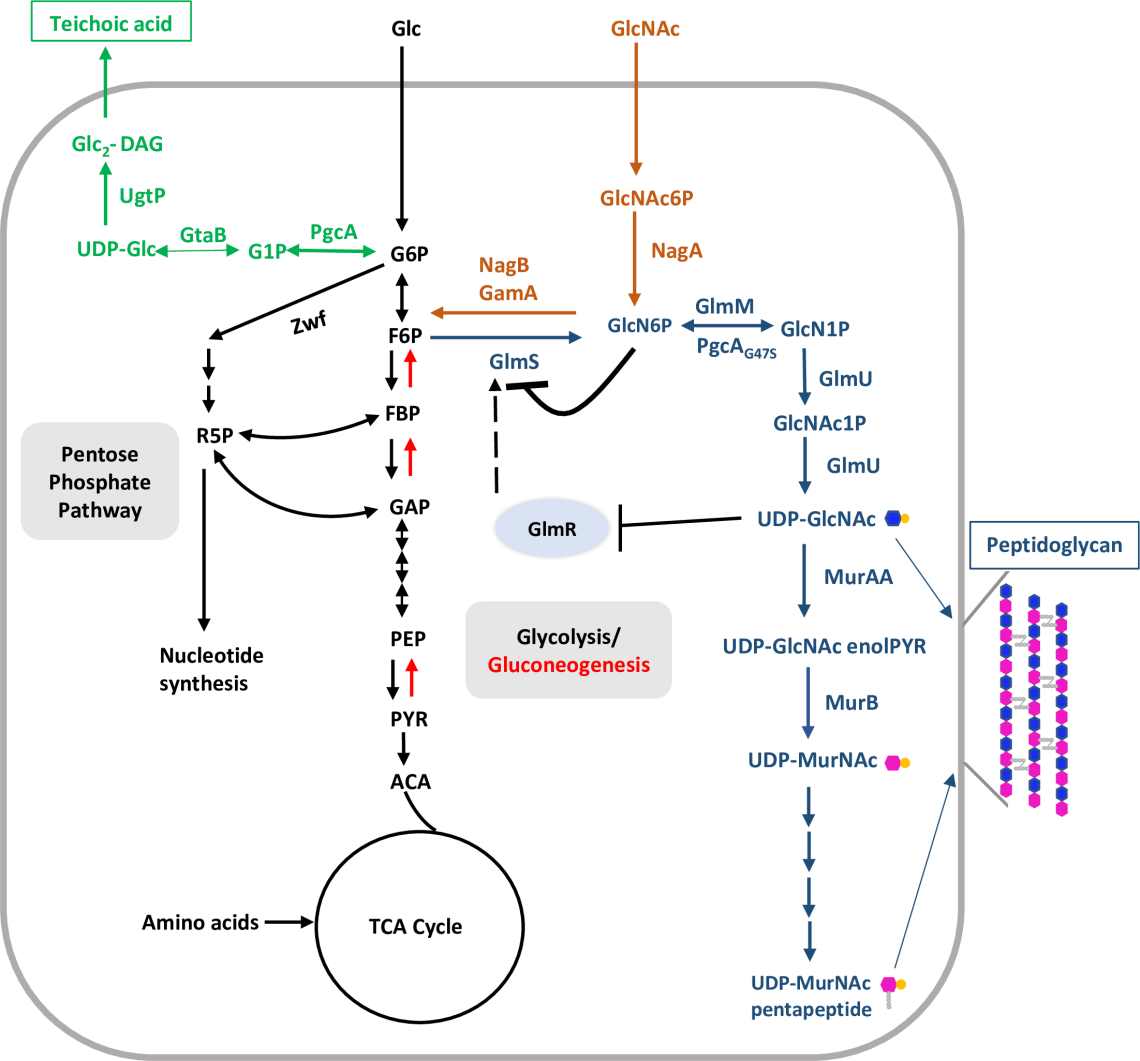
Schematic illustration of central carbon, peptidoglycan, UDP-Glc and UDP-GlcNAc metabolism. Central carbon metabolism (CCM; glycolysis/gluconeogenesis, pentose phosphate pathway and TCA cycle) is shown with black colored fonts and arrows. Black double headed arrows represent bidirectional enzyme reactions. Single headed black and red arrows represent glycolysis- and gluconeogenesis-specific enzymatic steps, respectively. UDP-Glc biosynthesis and its incorporation in teichoic acids is depicted in green. Steps of peptidoglycan biosynthesis and GlcNAc feeding into central carbon metabolism are shown in blue and orange, respectively. Black dashed arrow indicates activation.

**Fig 2:**
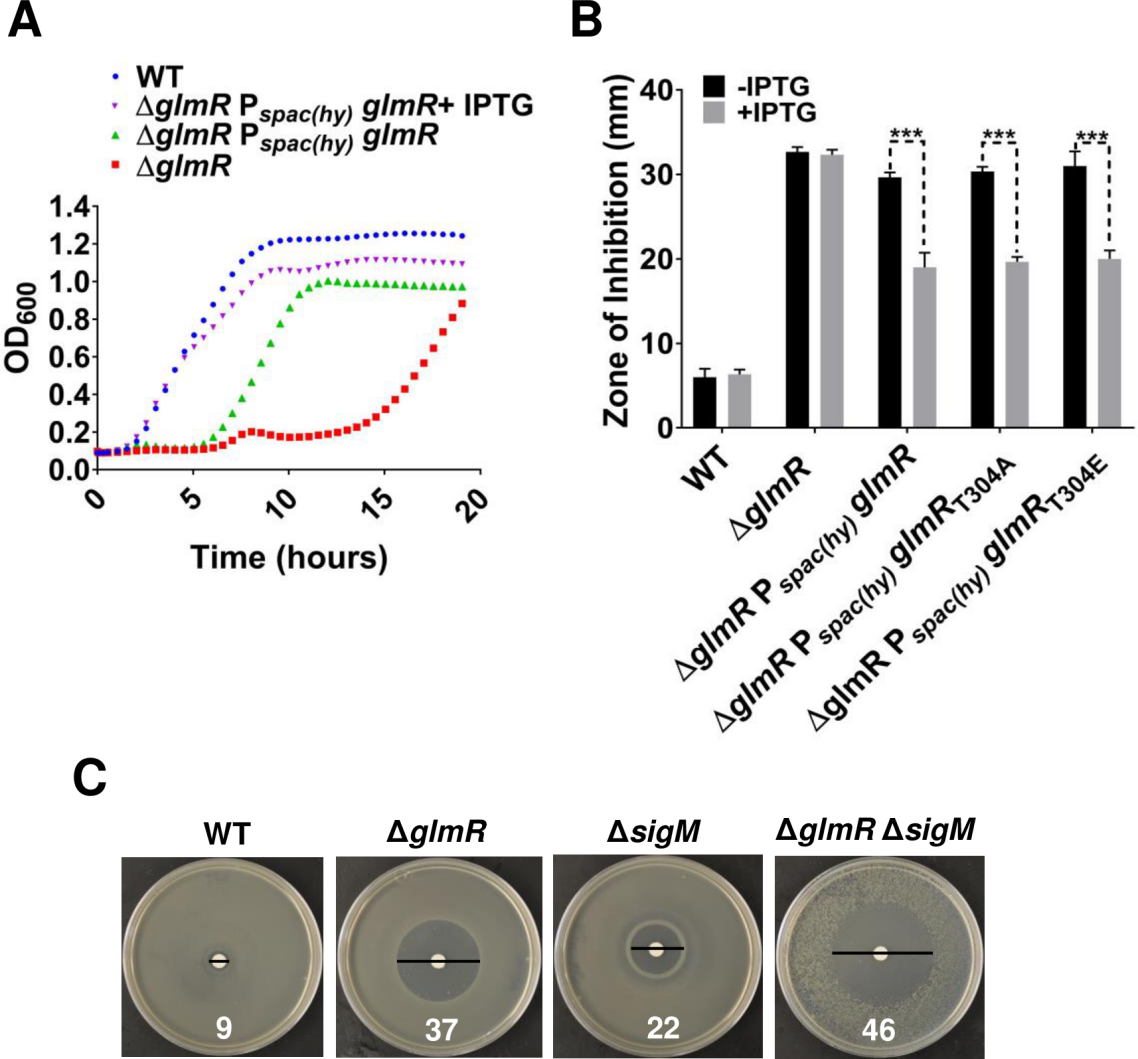
Δ*glmR* is unable to grow on MH medium and is sensitive to CEF. **(A)** Growth curves of WT, Δ*glmR* and Δ*glmR amyE*:: P_spac-(hy)_ *glmR* on gluconeogenic MH media. **(B)** CEF susceptibility of Δ*glmR* and complementation of the phenotype was tested by disc diffusion assay using 6 μg of antibiotic. IPTG was added to 1 mM to induce expression of ectopic copy of *glmR*. Zone of inhibition (ZOI) was measured after overnight incubation of plates at 37°C. ZOI represents the diameter of clear zone surrounding the disc minus the disc (7 mm). Standard deviation (error bars) is based on at least three biological replicates. Three asterisks indicate significant difference with P<0.001 estimated by comparing IPTG treated samples with untreated samples using Tukey test. **(C)** Evidence that *glmR* functions independently of *sigM*. Epistasis between *glmR* and *sigM* was determined by disc diffusion assay with 3 μg of CEF on each filter.

To test the role of GlmR in the connection between CCM and PG biosynthesis (Fig 1), we generated a *B*. *subtilis* strain with an in-frame, unmarked deletion of *glmR* (Δ*glmR*) and characterized its growth properties and sensitivity to cell wall antibiotics. Mueller-Hinton (MH) is a gluconeogenic medium containing amino acids as primary carbon source and is commonly used for antibiotic sensitivity experiments. However, Δ*glmR* is unable to grow on MH. This phenotype can be complemented by an ectopic, inducible copy of *glmR* (Fig 2A) or addition of glucose (S1A and S1B Fig), consistent with prior results [5].

To monitor the impact of the Δ*glmR* mutation on antibiotic sensitivity we performed zone-of-inhibition assays using LB (lysogeny broth) medium, a complex medium containing a variety of mono- and disaccharides (a total carbohydrate concentration of ~0.16%; [14]) and abundant amino acids. The Δ*glmR* mutant is much more sensitive to the beta-lactam antibiotic cefuroxime (CEF) (Fig 2B) as well as to other beta-lactam antibiotics (oxacillin and cefixime), moenomycin, and vancomycin (S2A–S2D Fig), all of which act by affecting the assembly and cross-linking of the peptidoglycan sacculus. However, we did not observe any significant difference in susceptibility between wild-type (WT) and Δ*glmR* to fosfomycin, bacitracin or nisin (S2E–S2G Fig). The lack of significant effect with these compounds may be due to the presence of inducible resistance mechanisms that might mask the effects of the Δ*glmR* mutation [15–18].

We selected CEF for further study due to the significantly higher sensitivity of the Δ*glmR* strain. Induction of an ectopic, IPTG-inducible *glmR* gene partially complements Δ*glmR* cefuroxime sensitivity (Fig 2B). Incomplete complementation may indicate that GlmR levels from this construct, while sufficient to restore growth (Fig 2A), are insufficient for robust cell wall synthesis. Consistent with this idea, induction of an N-terminally 3X-FLAG-tagged *glmR* allele with an optimized ribosome-binding site (AGGAGG-seven base pairs upstream from start codon), complemented CEF resistance to WT levels (S3A Fig). Mutations affecting PG synthesis can often be suppressed by high concentrations of Mg^2+^ [19, 20]. Indeed, Mg^2+^ suppresses the growth defect of a *glmR* deletion mutant on non-glycolytic carbon sources (S1A Fig), as shown previously [5], and also partially suppresses CEF sensitivity (S3B Fig). These results suggest that a Δ*glmR* strain is impaired in PG synthesis, and therefore more susceptible to antibiotics that interfere directly with PG assembly such as beta-lactams.

Both the Δ*glmR* and Δ*sigM* mutants are CEF sensitive, and in both cases mutations known to increase c-di-AMP levels suppress this sensitivity (see below). This suggests that GlmR and σ^M^ may function in the same pathway. However, a Δ*glmR* Δ*sigM* double mutant is much more sensitive than either single mutant (Fig 2C), suggesting that these are two independent (and additive) pathways for intrinsic CEF resistance.

### The role of GlmR in intrinsic CEF resistance is phosphorylation independent

The CEF sensitivity of the Δ*glmR* strain is suggestive of a defect in PG synthesis. GlmR is also known to be modified on Thr304 by the penicillin binding protein and serine/threonine associated (PASTA) kinase PrkC and phosphatase PrpC [21]. PrkC is activated by muropeptides during spore germination [22] and is regulated by interaction with the cell division protein GpsB during growth [23]. PrkC-dependent phosphorylation of GlmR has been linked to its role in morphogenesis and to resistance to bacitracin, but appears not to be required for growth on gluconeogenic carbon sources [21]. Similarly, this post-translational modification is not required for suppression of CEF sensitivity: both the phosphomimetic GlmR_T304E_ and phosphoablative GlmR_T304A_ mutant proteins complement the null mutant as well as wild-type (Fig 2B).

### Many Δ*glmR* suppressor mutations affect the *cdaA-cdaR-glmM-glmS* operon

To gain insight into the role of GlmR in *B*. *subtilis*, we characterized suppressors (both spontaneous and transposon-generated) that either increased CEF resistance or restored the ability of Δ*glmR* to grow on MH medium. We isolated CEF resistant Δ*glmR* suppressors from CEF zone-of-inhibition assays or as colonies on MH medium (S1B Fig). We identified the causative mutations using whole-genome resequencing (spontaneous mutations) or by sequencing of junction fragments (transposon insertions) followed by linkage analysis and/or genetic reconstruction and complementation (Table 1). In general, the selected mutations suppressed both phenotypes associated with Δ*glmR*. Those suppressors selected for increased CEF resistance also recovered an ability to grow on MH medium. Conversely, for those selected for growth on MH medium, nearly all displayed at least a partial increase in CEF resistance relative to the Δ*glmR* starting strain (Table 1). In general, in this and previous studies, we find that CEF sensitivity is an excellent reporter for defects in cell wall synthesis. Often, suppressor mutations that fully restore growth may only partially rescue intrinsic CEF resistance. Here, we will focus on those suppressor mutations in the *cdaA-cdaR-glmM-glmS* region of the chromosome, which encodes the two initial enzymes in the peptidoglycan biosynthesis pathway, a major cyclic-di-AMP synthase (CdaA) and a regulator of CdaA (CdaR). We also recovered mutations in other genes in carbon metabolism, including *pgcA* and *zwf*, consistent with prior genetic studies of *glmR* function [5]. The possible mechanisms of suppression for these and other mutations are considered in the Discussion.

**Table 1:** Δ*glmR* suppressor mutations.

Selection	Mutant	Genomic region changes	Coding region change	Affected gene or non-coding area	Gene annotation	CEF^R^ (ZOI, mm)	Growth on MH	Linkage
CEF^R^	*glmS1*	200068A>T		Inside *glmS* ribozyme	Senses GlcN6P and controls expression of *glmS*	31	Yes	Yes
	*rsiW1*	196049G>A	E208E	Penultimate codon of *rsiW* (E208E), affects termination loop stability	Anti-*sigW*	27	Yes	Yes
	*rsiW2*	196071C>T		Located downstream of *rsiW*. Affects termination loop stability	Non-coding region	27	Yes	Yes
	*rsiW3*			Tn insertion downstream of rsiW stop codon	Non-coding region	35	ND	Yes
	*pgcA*_G47S_	1006912G>A	G47A	*pgcA*	Phosphoglucomutase	30	Yes	Yes
	*yvcJ*_L104H_ *sigA*_A197V_	3572078T>A 2600750G>A	L104H A197V	*yvcJ* *sigA*	GTPase, nucleotide-binding protein; primary σ factor	31	ND	ND
	*tufA1*::*TnYLB-1*			Tn insertion downstream of *tufA* after stop codon	Elongation factor Tu	29	ND	Yes
MH	*glmS1*	200068A>T		Inside *glmS* ribozyme	Senses GlcN6P and controls expression of *glmS*	31	Yes	Yes
	*zwf*_D405fs_	2480369delA	D405stop	*zwf*	Glucose 6-phosphate dehydrogenase	31	Yes	Yes
	*ispA*_L140P_	2526261A>G	L140P	*ispA*	Geranyltransferase	39	Yes	ND
	*ispA*::*TnYLB-1*			Tn insertion in *ispA*	Geranyltransferase	35	Yes	Yes
	*clpX*::*TnYLB-1*			Tn insertion in *clpX*	ATP-dependent Clp protease	19	Yes	Yes
	*qoxB*::*TnYLB-1*			Tn insertion in *qoxB*	Cytochrome aa3 quinol oxidase	26	Yes	Yes

**Table 1:** List of Δ*glmR* suppressors obtained using CEF resistance (CEF^R^) or growth on MH medium as selection. CEF sensitivity was selected starting with a Δ*glmR* strain (a zone-of-inhibition, ZOI = 40 mm). For comparison, WT has a ZOI = 12 mm. Genomic region change indicates location of a nucleotide on reference genome of *B*. *subtilis* subsp. 168 (NCBI reference sequence NC_000964.3). Coding region changes show predicted amino acid substitutions.

**Fig 3:**
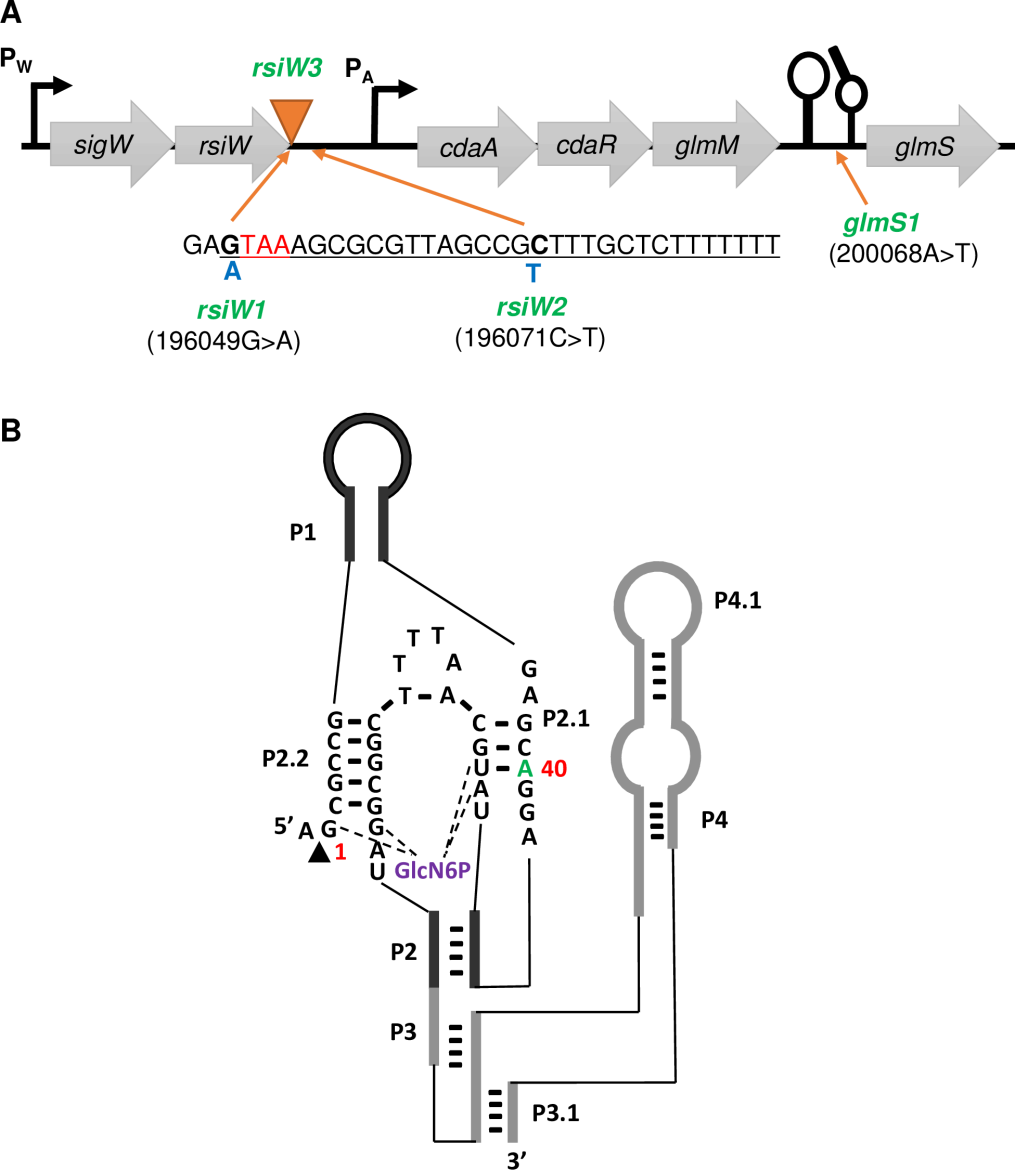
Location of *glmR* suppressor mutations in the *sigW-rsiW* and *cdaA-cdaR-glmM-glmS* operons. **(A)** The *sigW-rsiW* transcription termination loop (underlined) is shown with the *rsiW* stop codon (red). Suppressor mutations included single nucleotide changes (*rsiW1* and *rsiW2*; blue) and a transposon insertion (*rsiW3*; orange triangle). **(B)** Secondary structure of *glmS* ribozyme catalytic domain in *B*. *subtilis*. The arrowhead indicates the site of self-cleavage. The guanine at the cleavage site is considered the first residue (G1). The green letter (40A) identifies the site of the *glmS1* mutation (40A>T).

Many of the Δ*glmR* suppressors (Table 1) contained changes in a chromosomal region around two neighboring operons: *sigW-rsiW* and *cdaA-cdaR-glmM-glmS* (Fig 3A). These included a transposon insertion immediately after the *rsiW* stop codon (*rsiW3*) and point mutations in the *glmS* ribozyme (*glmS1*; 200068A>T), in the penultimate codon of *rsiW* (*rsiW1*; 196049G>A), and downstream of *rsiW* (*rsiW2*; 196071C>T). Note that the identical *glmS* mutation (*glmS1*) was recovered independently in both selection conditions.

Since most of the suppressor mutations did not fully restore CEF resistance to WT levels (Table 1), we selected several with intermediate levels of resistance as a starting point for selection of further increased CEF resistance. The most frequent secondary mutations were in *rho* (S1 Table). A *rho* deletion mutant has been associated with beta-lactam resistance in *B*. *subtilis* previously [24]. Interestingly, a Δ*glmR* Δ*rho* double mutant is actually more sensitive to CEF than Δ*glmR* (S4 Fig), and it is only when a primary suppressor mutation (such as *glmS1*) is present in Δ*glmR* that *rho* mutations confers significant CEF resistance (S4 Fig and S1 Table).

### The *glmS1* ribozyme mutation abolishes negative feedback regulation of *glmS*

GlmS is an amidotransferase that catalyzes the first step in PG synthesis (Fig 1) by conversion of the glycolysis intermediate fructose-6-phosphate (F6P) into glucosamine-6-phosphate (GlcN6P) using glutamine as an amino group donor [25]. Expression of GlmS is under negative feedback control mediated by a ribozyme structure encoded in the 5'-untranslated region (5’-UTR) of the *glmS* mRNA. Upon binding to the GlmS product, GlcN6P, the ribozyme promotes site specific self-cleavage of *glmS* mRNA and consequently reduces *glmS* expression [26].

**Fig 4:**
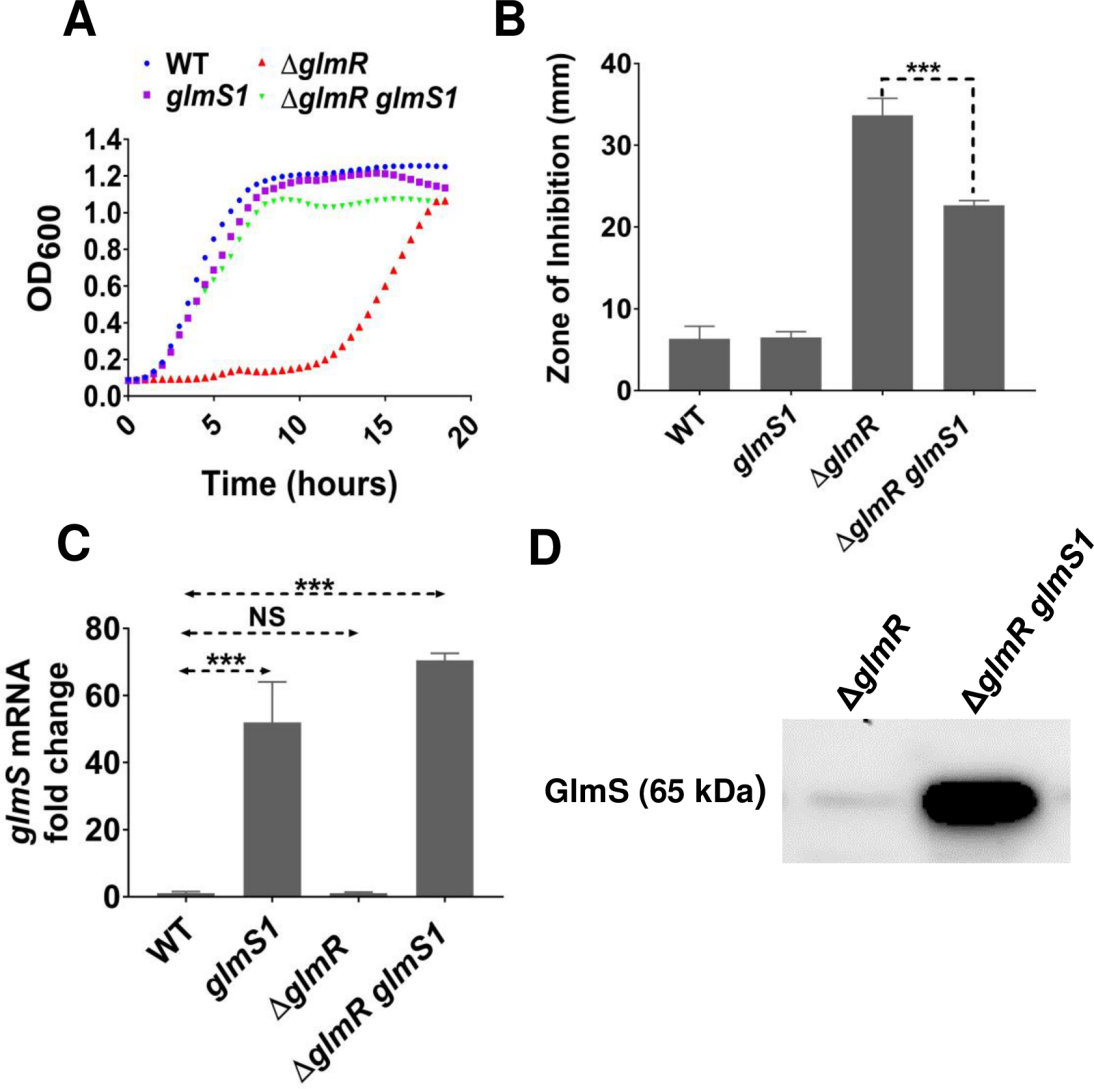
*glmS1* suppresses Δ*glmR* by abolishing negative feedback regulation of *glmS* expression. **(A)** Representative growth curves in MH medium (n>3) showing the effect of point mutation *glmS1* in Δ*glmR* compared to WT, *glmS1* and Δ*glmR*. **(B)** Disc diffusion assay showing the effect of *glmS1* on CEF sensitivity phenotype of Δ*glmR*. 6 μg CEF was used in the assay. Standard deviation (error bars) is based on at least three biological replicates. Three asterisks indicate significant difference with P <0.001 using Tukey test. **(C)** qRT-PCR results showing *glmS* mRNA level in *glmS1*, Δ*glmR* and Δ*glmR glmS1* relative to WT from cells harvested at OD_600_ of 0.5 grown in LB. Standard deviation (error bars) is based on at least three biological replicates. Statistical significance is determined by Tukey test where three asterisks indicate P <0.001 and NS is non-significant (P >0.05). **(D)** Western blot analysis of GlmS protein in Δ*glmR* and Δ*glmR glmS1* using anti-GlmS antibodies. 5 μg of total protein was loaded in each lane.

The *glmS1* suppressor mutation is a base change in the catalytic domain of the *glmS* ribozyme (Fig 3B) [27]. After moving the *glms1* mutation into a Δ*glmR* strain, the reconstructed Δ*glmR* *glmS1* strain regains the ability to grow on gluconeogenic carbon sources (Fig 4A) and has increased resistance to CEF (Fig 4B). We hypothesized that *glmS1* might interfere with the catalytic activity of the *glmS* ribozyme. Consistent with this idea, the *glmS1* mutation caused a >50-fold increase in *glmS* mRNA compared to WT (Fig 4C) and a corresponding increase in GlmS protein levels (Fig 4D). We did not see any significant difference in *glmS* mRNA level between WT and Δ*glmR*.

### Point mutations in the *sigW-rsiW* transcription termination loop suppress Δ*glmR*

**Fig 5:**
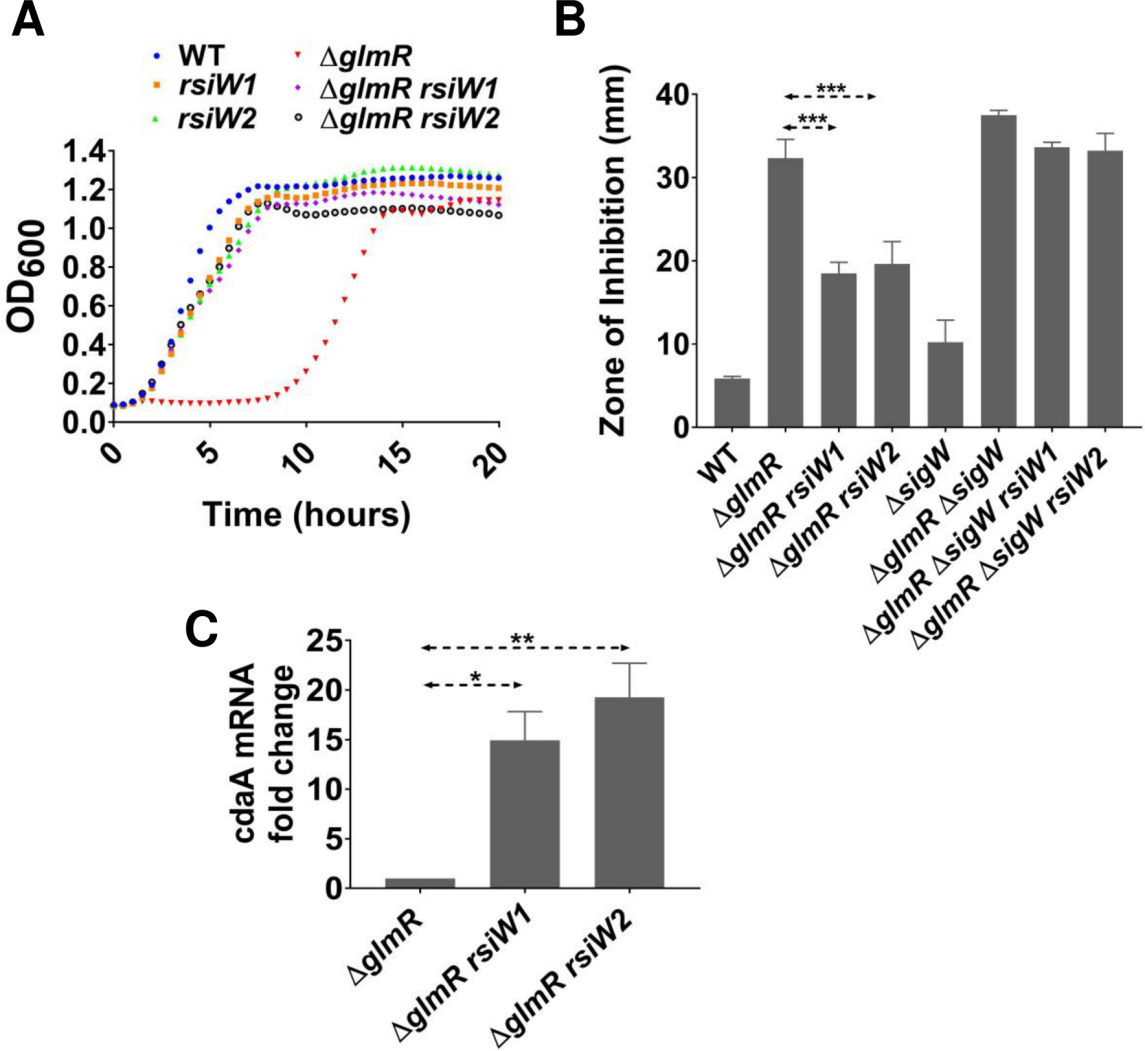
Point mutations in the *sigW-rsiW* operon transcription termination loop suppress Δ*glmR* phenotypes. **(A)** Growth curves showing the effect of *rsiW1* and *rsiW2* on the ability of Δ*glmR* to grow on gluconeogenic MH medium. (**B)** Disc diffusion assays carried out with 6 μg CEF. Standard deviation (error bars) is based on at least three biological replicates. Three asterisks represent statistical significance with P <0.001 with the Tukey test. **(C)** qRT-PCR analysis of *cdaA* mRNA fold change relative to *glmR*. One and two asterisks represent statistical significance with P value less than 0.05 and 0.01 respectively.

Reconstruction of Δ*glmR* strains with mutations *rsiW1* or *rsiW2* confirmed that these changes allow growth of Δ*glmR* on gluconeogenic growth medium (Fig 5A) as well as increased resistance to CEF (Fig 5B). The *rsiW1* mutation is silent with respect to the sequence of RsiW and *rsiW2* is downstream of the *rsiW* coding region (Fig 3A). We hypothesized that these point mutations might affect the intrinsic transcription terminator of the *sigW-rsiW* operon. *In silico* analysis indicated that each mutation generates a mismatch in the stem of the transcription terminator that is predicted to decrease stability and therefore increase readthrough from the *sigW-rsiW* operon into the downstream *cdaA-cdaR-glmS-glmM* operon (S5 Fig). Indeed, the *rsiW1* or *rsiW2* suppressor mutations led to a >10-fold increase in the mRNA level for the first gene of this operon, *cdaA* (Fig 5C).

Expression of the *sigW-rsiW* operon is dependent on an autoregulatory σ^W^-dependent promoter. An in-frame deletion mutation of *sigW* abolished the ability of the *rsiW1* and *rsiW2* mutations to suppress the Δ*glmR* phenotype (Fig 5B). However, in a strain with a *sigW*::*erm* disruption mutation the *rsiW1* and *rsiW2* mutations still conferred increased CEF resistance since the *erm* σ^A^ promoter now reads into the *cdaA* operon (S5B Fig). These observations support our hypothesis that *rsiW1* and *rsiW2* increase expression of *cdaA-cdaR-glmM-glmS*. A similar increase in transcription may explain the phenotype of the *rsiW3* Tn insertion (Table 1).

### Increased expression of genes from the *cdaA-cdaR-glmM-glmS* operon suppresses Δ*glmR* growth phenotypes

**Fig 6:**
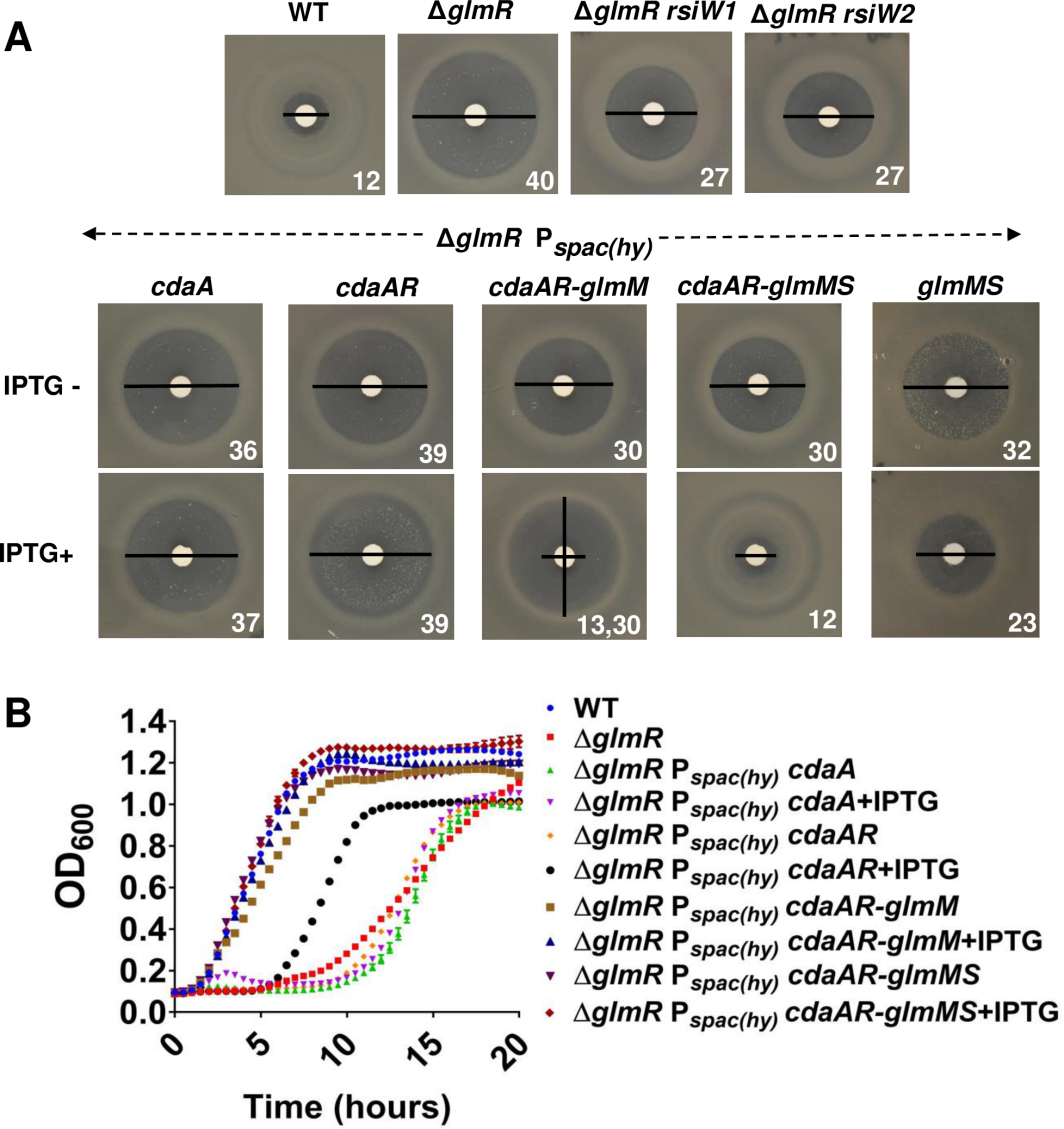
Suppression of *glmR* by overexpression of *glmS* and *glmM*. **(A)** Disc diffusion assays (representative images; n>3) illustrating effects of overexpression of *cdaA*, *cdaA-cdaR (cdaAR)*, *glmM*-*glmS*, *cdaAR-glmM* or *cdaAR-glmM-glmS* on the CEF sensitivity of the Δ*glmR* strain. Numbers represents diameter of ZOI (mm). Note that for *cdaAR-glmM* there is a small clear inner zone (13 mm), and a larger zone of greatly reduced growth (30 mm). **(B)** Growth curves in MH media for the strains shown in panel (A).

We reasoned that the *rsiW1*, *rsiW2* and *rsiW3* mutations likely lead to elevated expression of the *cdaA-cdaR-glmM-glmS* operon. The first two genes encode the major synthase (CdaA) for c-di-AMP and an activator protein (CdaR) [6, 7]. The final two genes encode enzymes for the initial steps of PG biosynthesis that (together with GlmU; also known as GcaD; [28]) convert F6P to UDP-GlcNAc (Fig 1). To determine which gene(s) in this operon are involved in suppression of the Δ*glmR* phenotypes we integrated IPTG-inducible copies of various portions of this operon (including *cdaA*, *cdaA-cdaR*, *cdaA-cdaR-glmM*, *cdaA-cdaR-glmM-glmS*, *glmM-glmS*) at the *amyE* locus in the Δ*glmR* strain. These strains were tested for CEF sensitivity and growth on MH medium. Overexpression of *cdaA* or *cdaA-cdaR* was not sufficient to increase CEF resistance of Δ*glmR* (Fig 6A), although we did note an increased frequency of spontaneous suppressors. Overexpression of *cdaA-cdaR-glmM* or *glmM-glmS* partially restored CEF resistance (Fig 6A). However, when the whole operon (*cdaA-cdaR-glmM-glmS*) was induced CEF resistance was restored to essentially WT levels (Fig 6A). Increased expression of *cdaA-cdaR-glmM* or *cdaA-cdaR-glmM-glmS* also suppressed the essentiality of Δ*glmR* on gluconeogenic MH medium (Fig 6B). In contrast, induction of *cdaA-cdaR* alone has a comparatively weak and variable effect on growth, which may reflect the rapid emergence of suppressors in this strain (Fig 6B). From these results we conclude that the key factor in increased fitness of the Δ*glmR* strain is elevated expression of GlmS and/or GlmM, but that c-di-AMP may also play a role.

An increase of c-di-AMP has been previously associated with CEF resistance since mutations in *gdpP*, encoding the major c-di-AMP hydrolase, suppress the CEF sensitivity of a *sigM* mutant [4]. Moreover, a *yqfF*::*Tn* insertion, affecting a second c-di-AMP hydrolase renamed PgpH [6, 7], suppresses the inability of a *glmR*(*yvcK*) mutant to grow on gluconeogenic media [5]. We have confirmed these findings and here demonstrate that inactivation of *gdpP* increases CEF resistance of Δ*glmR*, although *pgpH* does not have a significant effect under our conditions (S6A and S6B Fig). It is interesting to note that a *gdpP pgpH* double mutant, which has greatly elevated c-di-AMP levels and is growth impaired [7], is also highly sensitive to CEF. This effect is not additive with Δ*glmR*, suggesting that excess c-di-AMP may affect the same pathway as GlmR (S6A and S6B Fig). Consistently, the ability of CdaA and CdaR to increase CEF resistance in a Δ*glmR* mutant seems to be contingent on the additional expression of GlmM and GlmS, as noted above (Fig 6A). CdaA forms a complex with both CdaR and GlmM [7, 29], suggesting that c-di-AMP may modulate GlmM activity.

### Increasing expression of UDP-GlcNAc biosynthetic enzymes suppresses Δ*glmR* phenotypes

**Fig 7:**
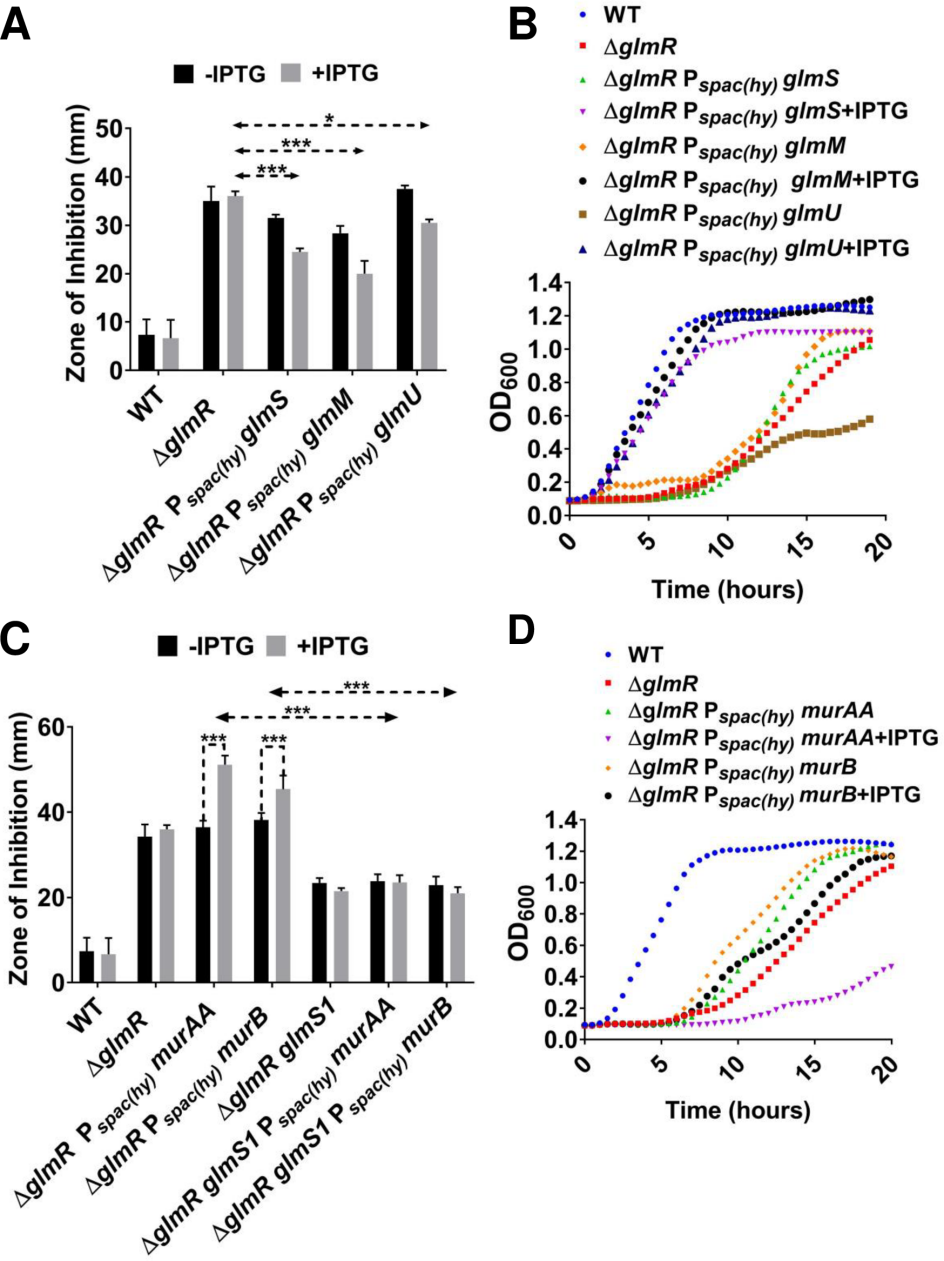
Increasing UDP-GlcNAc suppresses Δ*glmR*. **(A)** Disc diffusion showing the change in CEF susceptibility of Δ*glmR* when *glmM*, *glmS* and *glmU* were overexpressed. Standard deviation (error bars) is based on at least three biological replicates. One and three asterisks indicate significant value with P <0.05 and P <0.001 respectively as determined by Tukey test. **(B)** Growth curves in MH media with Δ*glmR* overexpressing *glmM*, *glmS and glmU* in comparison to WT and Δ*glmR*. **(C)** Disc diffusion assay showing CEF susceptibility of Δ*glmR* when *murAA* and *murB* are overexpressed. The figure also shows the effect on CEF sensitivity when *glmS1* is introduced in Δ*glmR amyE*:: P_spac(hy)_ *murAA* and Δ*glmR amyE*::P_*spac(hy)*_ *murB* respectively. Three asterisks indicate significance (P <0.001) as determined by Tukey test. **(D)** Growth curve experiment done in MH medium showing the consequence of *murAA* and *murB* overexpression on Δ*glmR*.

We next considered whether a Δ*glmR* strain might be phenotypically suppressed by over-expression of other individual enzymes upstream and downstream of UDP-GlcNAc. Induction of *glmS*, *glmM* or *glmU* (Fig 1), partially restored CEF resistance (Fig 7A) and restored the ability of Δ*glmR* to grow on gluconeogenic medium (Fig 7B). We suggest that these enzymes increase the forward reaction catalyzed by GlmS by consumption of the product, GlcN6P. GlcN6P is potent inhibitor of GlmS (product inhibition) [30], a property shared with the human ortholog [31].

A portion of cellular UDP-GlcNAc is converted to UDP-MurNAc, the second building block of PG, by MurA and MurB (Fig 1). *B*. *subtilis* has two MurA paralogs, MurAA and MurAB, but only MurA is essential. UDP-MurNAc is then modified by addition of a pentapeptide side-chain and transferred to the undecaprenylphosphate carrier lipid to ultimately generate lipid II (Fig 1), a lipid-linked GlcNAc-MurNAc-pentapeptide that is the substrate for extracellular PG synthesis [32]. Overexpression of *murAA* or *murB* increased the sensitivity of the Δ*glmR* strain to CEF (Fig 7C), and neither rescued the growth defect of Δ*glmR* on MH medium (Fig 7D).

We reasoned that the effects of MurAA and MurB overproduction might be relieved in cells that have increased capacity to synthesize UDP-GlcNAc. To test this hypothesis, we introduced the *glmS1* mutation (which abolishes negative feedback regulation of *glmS*) into the Δ*glmR amyE*::P_spac(hy)_ *murAA* and Δ*glmR amyE*::P_spac(hy)_ *murB* strains. In these *glmS1* strains, induction of *murAA* or *murB* no longer increases sensitivity to CEF (Fig 7C). Based on these observations we hypothesize that *B*. *subtilis* lacking GlmR is impaired specifically in UDP-GlcNAc biosynthesis. The resulting inability to efficiently synthesize PG is a likely reason for the essentiality of *glmR* on gluconeogenic media.

### Mutations of the GlmR UDP-GlcNAc binding site do not significantly affect CEF resistance

**Fig 8:**
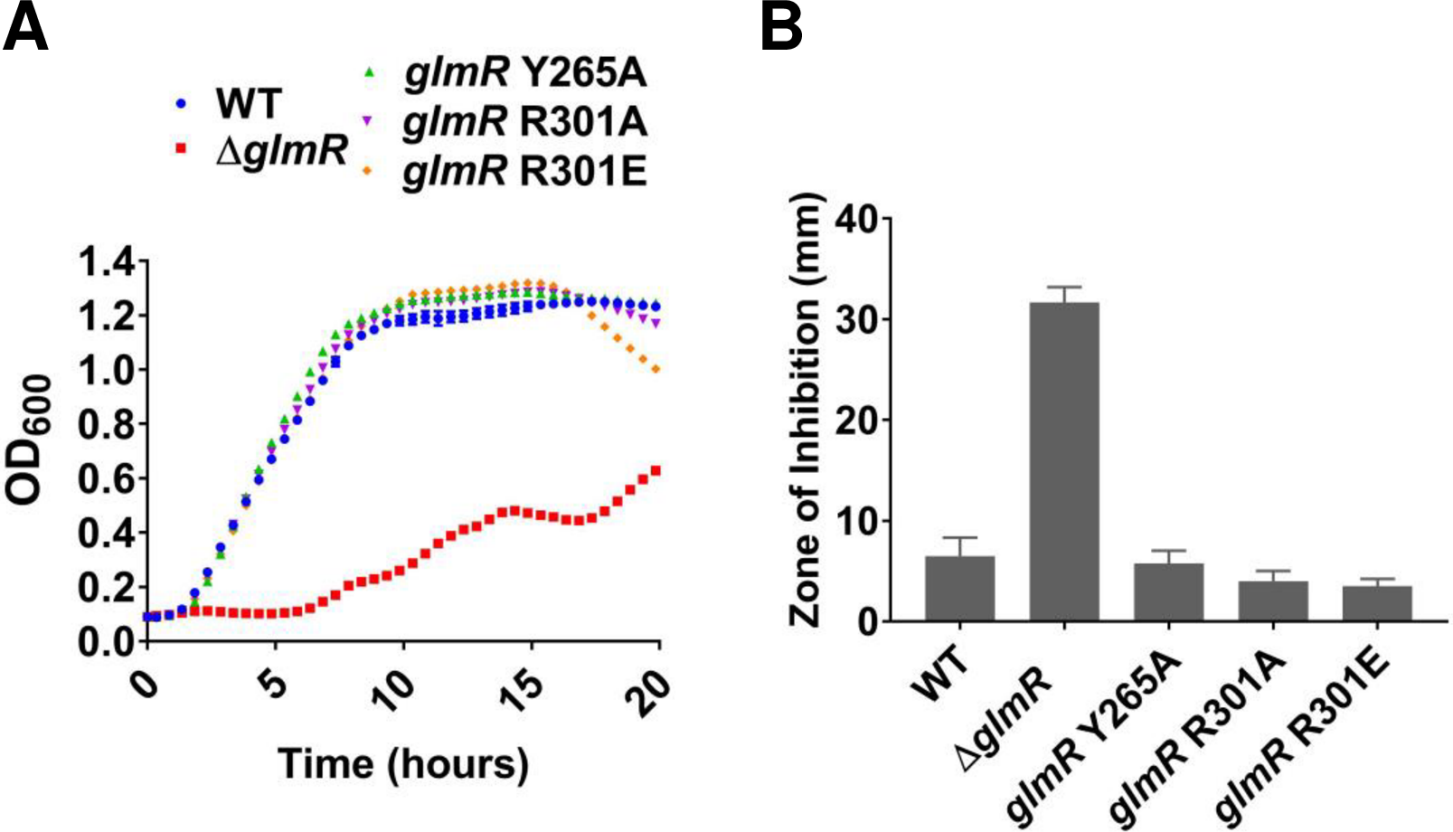
Mutations of the GlmR UDP-GlcNAc binding site do not affect gluconeogenic growth. **(A)** Growth curve with UDP-GlcNAc binding site mutants of GlmR in gluconeogenic MH medium and **(B)** CEF sensitivity of GlcNAc binding site mutants of GlmR tested on LB medium with 6 μg of antibiotic.

GlmR was recently found to bind UDP-sugars such as UDP-glucose and UDP-GlcNAc [10]. UDP-GlcNAc bound with five times higher affinity that UDP-Glc, suggesting that the former may be a regulatory ligand for GlmR. We used CRISPR-gene editing to introduce single amino acid substitutions in the UDP-GlcNAc binding site of GlmR that were previously shown to abolish ligand binding (Y265A, R301A and R301E). Consistent with prior results [10], none of these three mutations affected the ability of GlmR to support growth on gluconeogenic MH medium (Fig 8A), nor did they have a significant impact on CEF resistance (Fig 8B). We therefore suggest that ligand binding serves as a feedback mechanism to down-regulate GlmR activity when UDP-GlcNAc levels are high. Under gluconeogenic conditions, when GlmR is required for redirecting carbon from CCM into PG synthesis, this binding site would be vacant, and therefore these mutations would not affect the stimulatory function of GlmR (Fig 1).

### Addition of GlcNAc bypasses the essentiality of *glmR* on gluconeogenic media

**Fig 9:**
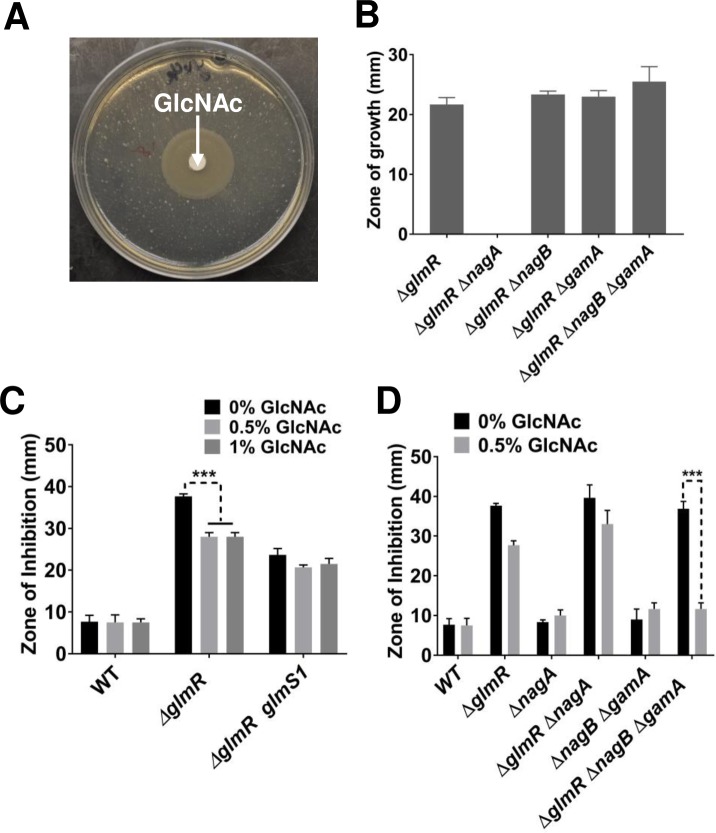
Addition of GlcNAc bypasses the essentiality of *glmR* on gluconeogenic media. **(A)** An MH agar plate with Δ*glmR* showing the zone of growth around a GlcNAc disc. **(B)** Bar graphs representing the zone of growth for Δ*glmR*, Δ*glmR* Δ*nagA*, Δ*glmR* Δ*nagB*, Δ*glmR* Δ*gamA* and Δ*glmR* Δ*nagB* Δ*gamA*. **(C)** Disc diffusion assay showing the effect of GlcNAc (0.5 or 1%) on CEF sensitivity of WT, Δ*glmR* and Δ*glmR glmS1* and **(D)** Disc diffusion assay comparing the effect of 0.5% GlcNAc on CEF sensitivity of WT, Δ*glmR*, Δ*nagA*, Δ*glmR* Δ*nagA*, Δ*gamA* Δ*nagB* and Δ*glmR* Δ*gamA* Δ*nagA*. Three asterisks indicate significance with P <0.001 as determined by Tukey test.

Since Δ*glmR* suppressor mutations lead to increased *glmS* expression (Fig 4C and 4D), we reasoned that the Δ*glmR* strain may be specifically defective in GlmS activity. If this is the case, we hypothesized that provision of cells with GlcNAc would chemically complement the Δ*glmR* growth defect. Indeed, when a disc containing GlcNAc was placed on a MH medium plate strong growth of the Δ*glmR* strain was observed (Fig 9A).

GlcNAc is taken up by the GlcNAc-specific phosphoenolpyruvate phosphotransferase system (PTS) protein NagP and enters the cell as GlcNAc-6-phosphate [33]. Deacetylation by NagA then generates GlcN6P (Fig 1), which is also the product generated by GlmS [34]. GlcN6P can either feed into peptidoglycan biosynthesis (GlmM and GlmU) or feed CCM by conversion to F6P by either of two inducible deaminases (NagB and GamA) [33, 35] (Fig 1). The ability of GlcNAc to support growth of the Δ*glmR* strain requires NagA, but is independent of the GamA and NagB deaminases (Fig 9B). This indicates that the limiting step in metabolism during growth of the Δ*glmR* strain on largely gluconeogenic carbon sources is the GlmS-catalyzed conversion of F6P to GlcN6P. This limitation can be by-passed by up-regulation of GlmS (e.g. by overexpression, Fig 7B, or in the *glmS1* mutant strain, Fig 4) or by provision of cells with GlcNAc. The ability of overproduced GlmM or GlmU to support growth (Fig 7B) may therefore seem surprising, but may be explained by more rapid consumption of GlcN6P, which would prevent product inhibition of GlmS and also increase translation of GlmS by inhibiting *glmS* ribozyme cleavage.

**Fig 10:**
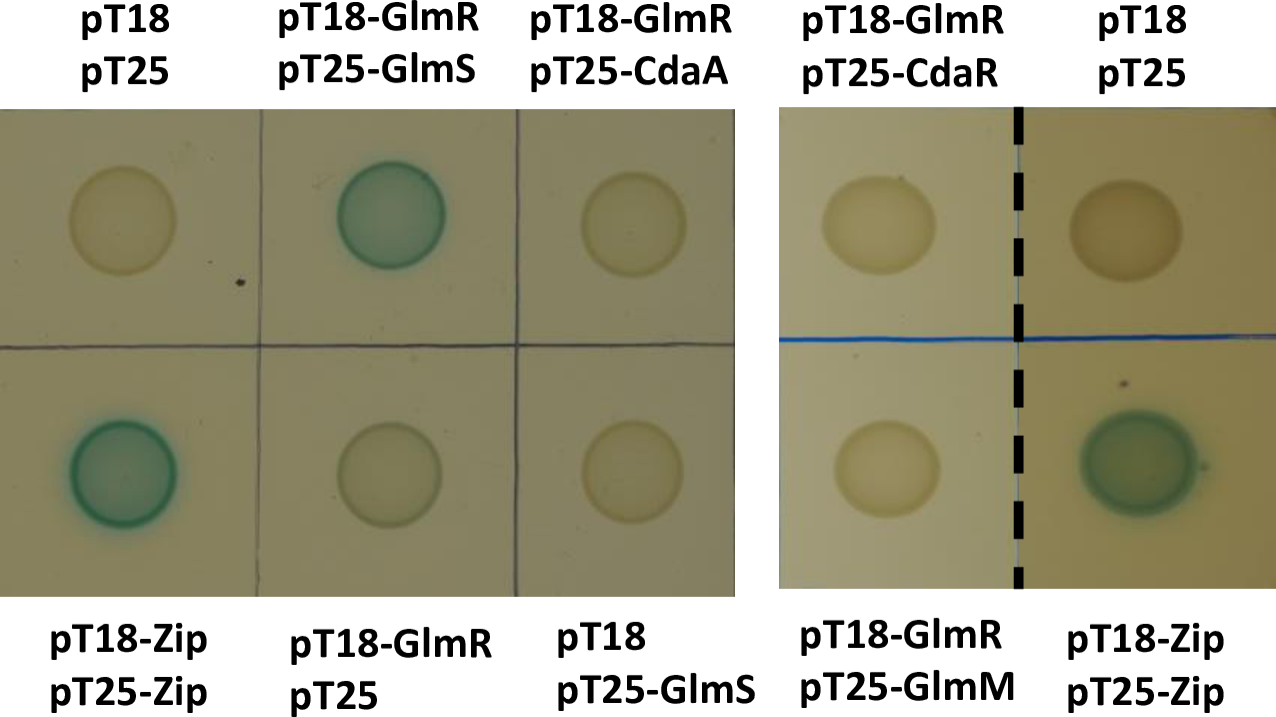
Bacterial two hybrid assay. pT18-containing *glmR* and the compatible plasmid pT25 containing *glmS* or *cdaA* were transformed into *E*. *coli* strain BTH101. When co-expressed protein fusions interact, the *Bordetella pertussis* adenylate cyclase is active as assessed by blue color in the presence of X-gal. Negative controls include cells containing one or both empty vectors, and the positive control is the pT18-ZIP and pT25-ZIP plasmids [60]. Left panel is a single plate, and the right panel is images from a separate experiment showing a lack of signal for interaction of GlmR with CdaR or GlmM and the corresponding positive control.

To test if GlcNAc addition also suppresses the increased CEF sensitivity, we tested WT and Δ*glmR* strains on LB agar supplemented with 0.5% and 1% GlcNAc. Addition of GlcNAc partially suppressed the CEF sensitivity of Δ*glmR*, but had no significant effect on a strain in which GlmS was up-regulated by the *glmS1* suppressor mutation (Fig 9C). In a Δ*glmR* Δ*nagB* Δ*gamA* strain in which added GlcNAc cannot re-enter CCM, CEF resistance is restored to near WT levels (Fig 9D). The greater suppression seen in this strain may result from the inability of this strain to catabolize incoming GlcNAc, which thereby further increases the flux into PG synthesis. This supports the notion that a major contributor to CEF sensitivity is a metabolic defect that limits the ability of the cell to synthesize PG, apparently due to a limitation in the ability of GlmS to redirect carbon from CCM to cell wall synthesis. We hypothesize that GlmR may directly stimulate GlmS enzyme activity. This is supported by evidence of a GlmR-GlmS protein interaction in bacterial two-hybrid assays (Fig 10). The observed interaction is robust, as compared to the positive control, and GlmR did not interact with other proteins tested including CdaA, GlmM or CdaR (Fig 10).

## Discussion

Here we present a forward genetic analysis that indicates that GlmR regulates the redirection of carbon from CCM into PG biosynthesis, likely by stimulation of GlmS activity. The regulation of CCM as cells adapt to nutrient availability is exceptionally complex and involves numerous transcriptional regulators and post-transcriptional regulatory mechanisms [36, 37]. The carbon catabolite control protein CcpA plays a central role in this process and represses genes for the utilization of non-preferred carbon sources when glucose is available [38], as well as the operon encoding *glmR*: *yvcI-yvcJ-glmR-yvcL-crh-yvcN* [39]. As a result, GlmR should be most abundant when CcpA activity is low. CcpA repressor activity is indirectly stimulated by elevated levels of fructose-1,6-bisphosphate present during growth on preferred carbon sources [40–42]. During growth on non-preferred, gluconeogenic carbon sources GlmR will be more abundant, consistent with its role in diverting carbon to PG synthesis under these conditions.

The GlmR (formerly YvcK) protein is conditionally essential and plays a poorly defined role in cell morphology and antibiotic resistance [10–12, 21]. Homologs in *M*. *tuberculosis* (CuvA) and *L*. *monocytogenes* (YvcK) appear to also play a role in helping maintain cell shape [11, 12]. GlmR was suggested to lead to a dysregulation of carbon metabolism since mutations affecting metabolic enzymes (e.g. Zwf) and CCM regulatory proteins (e.g. CggR) suppress the *glmR* null mutant and allow growth on gluconeogenic carbon sources [5]. Cytological evidence suggests that GlmR and CuvA localize to membrane sites associated with PG synthesis, and it has been noted that GlmR and MreB appear to functionally substitute for one another, perhaps in coordinating the assembly of PG biosynthetic complexes [8, 11]. Despite intensive study, the connection between these disparate phenotypes has been elusive. Here, we propose that several of these phenotypes can be explained by GlmR-dependent stimulation of the key branchpoint enzyme, GlmS.

It remains possible that, in addition to stimulation of GlmS activity, GlmR may have other functions. This is suggested by the observation that the role of GlmR in intrinsic CEF resistance is independent of protein phosphorylation as judged by the analysis of phosphomimetic and phosphablative mutants (Fig 2). In contrast, phosphorylation of GlmR was shown to affect bacitracin sensitivity and cell morphogenesis in an *mreB* mutant background [21]. Although the *M*. *tuberculosis* GlmR ortholog CuvA is also modified by phosphorylation by Ser/Thr PASTA kinases, this modification is not important for complementation of carbon source specific growth defects or for localization to sites of PG synthesis [11, 12], and perhaps regulates other functions. Analysis of phosphosite mutants of the *L*. *monocytogenes* GlmR ortholog suggests that a phosphomimetic variant is unaffected in metabolism and cell wall homeostasis, but is impaired in virulence [11, 12]. Further studies are needed to clarify how GlmR phosphorylation affects some, but not all, activities of this protein.

### A model for GlmR as a feedback inhibited activator of GlmS

Our genetic analysis supports a model in which GlmR activates GlmS, and we suggest that this activity is inhibited when GlmR is bound to the downstream metabolite, UDP-GlcNAc (Fig 1). This model is supported by several key observations. First, overproduction of GlmS, in either the *glmS1* mutant or by induction from an ectopic *glmS* gene, is sufficient to restore growth of the *glmR* null mutant on MH medium (Figs 4 and 7). Second, a *glmR* mutant can be chemically complemented by GlcNAc, even under conditions where GlcNAc cannot be routed into CCM (Fig 9). Since metabolism of GlcNAc generates GlcN6P, this addition specifically bypasses the GlmS reaction (Fig 1). Therefore, we suggest that GlmS (rather than GlmM or GlmU) is limiting the flux of carbon into PG in the Δ*glmR* strain. Third, GlmR and GlmS interact *in vivo* as judged by a bacterial two-hybrid assay (Fig 10). Fourth, previous metabolomics measurements indicate that F6P levels are ~16-fold lower during growth on gluconeogenic carbon sources when compared to glucose [13], consistent with the requirement for GlmR under these conditions (Fig 1). Fifth, GlmR was recently found to bind UDP-GlcNAc [10]. However, mutations that abolish binding do not affect the ability of GlmR to stimulate growth under gluconeogenic conditions [10] or to provide intrinsic CEF resistance (Fig 8), as predicted by the hypothesis that UDP-GlcNAc antagonizes GlmR function (Fig 1). GlmS is recognized as the key branch-point enzyme in bacteria for diverting carbon from CCM into PG synthesis, and in eukaryotes the GlmS ortholog diverts carbon into hexosamine synthesis. Both classes of enzyme are in some cases feedback regulated by UDP-GlcNAc [43–47]. Here, UDP-GlcNAc binding is proposed to antagonize GlmR function, and therefore reduce stimulation of GlmS.

In addition to GlmS, we also demonstrate that overproduction of either GlmM or GlmU, but not by enzymes downstream of the key intermediate UDP-GlcNAc, can suppress the *glmR* growth defect under gluconeogenic conditions. GlmS catalyzes a reversible reaction, and its product (GlcN6P) is a potent inhibitor of the forward reaction [30]. Moreover, GlcN6P binds to the *glmS* ribozyme to cleave the mRNA and suppress translation [26]. Therefore, we suggest that increasing the level of GlmM and/or GlmU likely helps pull the reaction in the forward direction and may also stimulate GlmS translation.

### GlmR activation of GlmS as a framework for understanding other suppressor mutations

With a defined model in hand, we can revisit the other suppressor mutations recovered both in our selection conditions (Table 1) and the studies of Görke *et al*. [5]. As noted previously, many of the mutations that suppress *glmR* affect CCM. We recovered a frameshift mutation in *zwf*, a gene also recovered in the previous transposon-based selection for *glmR* suppressors [5]. Normally, Zwf diverts a substantial fraction of glucose-6-phosphate from glycolysis into the pentose phosphate pathway [48]. We speculate that in the absence of Zwf there is increased flux leading to F6P, the GlmS substrate. We also recovered a mutation in *pgcA*, which encodes another branch point enzyme that uses glucose-6-phosphate. Previously, it was reported that a mutation in *cggR*, encoding the central glycolytic genes regulator, also suppresses *glmR* [5]. Since a *cggR* null mutant will have increased levels of several key enzymes that function in both glycolysis and gluconeogenesis [49], we speculate that this mutation alleviates the metabolic restriction in the *glmR* strain by increasing gluconeogenesis and therefore F6P levels.

A second class of mutations that increase the fitness of the Δ*glmR* strain are those that lead to elevated c-di-AMP levels. This was foreshadowed by the finding that a *pgpH* (formerly *yqfF*) mutation suppresses *glmR* [5]. In our studies, we find that *gdpP* suppresses *glmR* both for growth on MH medium and for CEF resistance, whereas *pgpH* has a lesser effect (S6 Fig). CdaA is regulated by interaction with the CdaR protein and also forms a complex with GlmM [7, 29]. Indeed, the *cdaA-cdaR-glmM* genes are co-transcribed in a wide variety of species, suggesting a functional connection. This has led to the suggestion that GlmM may regulate c-di-AMP synthesis [7, 29]. Conversely, CdaA may regulate GlmM. In this scenario, conditions that lead to elevated c-di-AMP may alter the CdaA-CdaR complex to favor a stimulatory interaction of CdaA with GlmM. Indeed, it is striking that induction of the entire *cdaARglmMS* operon fully restores CEF resistance to a *glmR* mutant (Fig 6), whereas this is not the case for the *glmR glmS1* strain (Fig 4) or for induction of *glmS* alone (Fig 7). Alternatively, c-di-AMP is also known to regulate potassium homeostasis by interaction with both protein and RNA (riboswitch) targets [50–53]. This c-di-AMP dependent osmolyte transport is important for maintaining turgor pressure in the cell and it has been proposed that perturbations of c-di-AMP metabolism can affect cell envelope integrity by increasing resistance against osmotic stresses [54].

A third class of suppressor mutations is in genes important for energy generation by the electron transport chain. These include mutations in *qoxB*, encoding cytochrome aa3 quinol oxidase, and *yqiD(ispA)*, encoding a geranyltransferase that is involved in synthesis of isoprenoid compounds including menaquinone, an electron carrier important for respiration (Table 1). Mutations in both of these loci have been previously associated with an increased ability of cells to survive the transition to L-forms that lack a peptidoglycan cell wall [55]. This observation led to a model in which a lethal consequence of cell wall defects is oxidative damage triggered by increased flux through the electron transport chain when carbon flux into peptidoglycan is eliminated [55]. Regardless of the precise mechanism, it is intriguing that mutations in these same genes were recovered as suppressors of Δ*glmR*.

Finally, we recovered one strain containing a missense mutation in *yvcJ* (Table 1), the gene immediately upstream of *glmR*. The role of YvcJ is unknown, but it has GTPase activity, affects phosphorylation of an uncharacterized cell component, and has an apparent role in natural competence [56, 57]. Since this strain contained an additional mutation in *sigA* (Table 1), further work is needed to determine the effect of the *yvcJ* mutation on CEF resistance. Curiously, mutants of the *E*. *coli* YvcJ homolog (RapZ; formerly YhbJ) lead to overproduction of GlmS [58]. RapZ appears to sense GlcN6P and regulates the processing and stability of a small RNA, GlmZ, that activates GlmS synthesis [46, 58, 59]. It is presently unknown whether YvcJ plays a related role in *B*. *subtilis*, perhaps by interacting either with GlmR or the *glmS* ribozyme.

In conclusion, the results presented here highlight the importance of the GlmS branch point in regulating the flow of carbon from CCM into PG synthesis. In eukaryotes, GlmS orthologs serve as the initiating enzyme for hexosamine biosynthesis, and are sensitive to both GlcN6P product inhibition [31] and feedback regulation by UDP-GlcNAc, which binds to the isomerase domain [43, 44]. In bacteria, GlmS is also subject to complex regulation at the level of both synthesis and activity [45–47]. In *B*. *subtilis*, GlmS is feedback inhibited by its immediate product, GlcN6P [30], which also activates the *glmS* ribozyme [26]. GlmR provides another layer of regulation. Our results support a model in which GlmR stimulates GlmS activity, and we propose that binding of UDP-GlcNAc may attenuate this stimulation.

## Methods

### Bacterial strains and growth conditions

*B*. *subtilis* strains used are derived from strain 168 (*trpC2*) (S2 Table). *E*. *coli* strain DH5α was used for cloning and strain BTH101 [60] for bacterial two hybrid experiments. Bacteria were cultured in LB broth. Strains with a *glmR* deletion mutation were cultured on LB with 20 mM MgSO_4_ unless specified otherwise. Antibiotics were added to growth media when required at the following concentrations: 100 μg/ml ampicillin, 30 μg/ml chloramphenicol for *E*. *coli*, 10 μg/ml kanamycin, 10 μg/ml chloramphenicol, 5 μg/ml tetracycline, 100 μg/ml spectinomycin and 1 μg/ml erythromycin with 25 μg/ml lincomycin (erm; macrolide-lincomycin-streptogramin B resistance).

### Cloning, transformation and strain construction

For cloning procedures, restriction digestion and ligation with T4 ligase was done as per manufacturer's instructions (NEB, USA). Plasmids were then transformed into competent DH5α cells [61]. Cloning was confirmed by polymerase chain reaction (PCR) followed by Sanger sequencing. *B*. *subtilis* transformation was carried out in minimal competence media with 12 mM MgSO_4_. DNA was added when cells reached OD_600_ of ~0.7–0.8. Generation of *B*. *subtilis* strains overexpressing gene(s) at *amyE* was achieved using pPL82 [62] carrying gene(s) of interest followed by transformation into the indicated *B*. *subtilis* recipient strain.

Bacillus knockout erythromycin (BKE) strains with various gene deletion mutations of *B*. *subtilis* were obtained from the *Bacillus* Genetic Stock Center (BGSC) [63]. Chromosomal DNA from each BKE strain was transformed into our lab strain *B*. *subtilis* 168. The erythromycin resistance cassette was removed using pDR244 [63], which produces Cre recombinase at the permissive temperature of 30°C, to generate in-frame deletions. pDR244 was transformed into *B*. *subtilis* strain at 30°C and plated on LB plates with spectinomycin. Colonies were picked after two overnight incubations and patched three successive times on LB plates incubated at the non-permissive temperature 42°C overnight. Strains were then patched on spectinomycin- and erythromycin-containing plates to confirm the absence of both markers. All the deletion mutants used in study are markerless deletions except Δ*rho* (*rho*::*erm*).

Single nucleotide mutations *glmS1*, *rsiW1* and *rsiW2* were reconstructed using the integration vector pMutin4 that has an *erm* resistance marker and *lacZ* [64]. A fragment of DNA with the mutation of interest was cloned into pMutin4 and confirmed with PCR and Sanger sequencing. The vector was transformed into *B*. *subtilis* where it integrated at locus by single crossover homologous recombination. Transformants were selected on plates with Erm and 40 μg/ml X-gal. After overnight incubation, a few blue color colonies were picked. Since pMutin4 integration is unstable, cells were grown without antibiotic selection three consecutive times with each time adding 1:100 dilution of cells from previous culture. Cells were then plated on LB plates with X-gal and white colonies were picked and sequenced to find those strains that retained the single nucleotide mutation of interest.

### Mariner transposon mutagenesis

Mariner transposon mutagenesis procedure was carried out in Δ*glmR* as described previously [65]. In brief, Δ*glmR* was transformed with the pMarA vector. The strain with pMarA was grown in 5 ml LB broth until mid-exponential phase and various dilutions of cells were plated on selection medium. In independent experiments CEF resistance and ability to grow on MH media were used as a selection.

### Spontaneous suppressor analysis

Spontaneous suppressors of Δ*glmR* were picked from the clear zone of CEF disc diffusion plates and independently from MH plates after overnight incubation at 37°C. Chromosomal DNA extracted from these suppressors was sequenced using an Illumina machine. The sequencing data were analyzed using CLC genomics workbench.

### Antibiotic sensitivity assays

Antibiotic sensitivity was tested using disc diffusion assays, which were carried out on LB medium. Strains to be tested were grown in 5 ml LB broth at 37°C with vigorous shaking to an OD_600_ of ~0.4. 100 μl of cells were added to 4 ml top LB agar (0.7% agar) kept at 50°C. 1 mM IPTG was added to top agar when indicated. Top agar with cells was poured over 15 ml LB bottom agar (1.5%) plate. A Whatman paper disc (7mm dia) with 6 μg CEF was put on the plate unless specified otherwise. Plates were incubated at 37°C overnight and the clear zone of inhibition was measured the next day. Values for CEF resistance (Table 1) report the diameter of the zone of growth inhibition. For all histograms, the values shown have the diameter of the filter disk (7 mm) subtracted from the average diameter.

### Growth Assay on MH

To test the ability of *B*. *subtilis* mutants to grow under gluconeogenic conditions we used MH medium (Sigma-Aldrich, USA) prepared per the manufacturer's instruction. Growth was monitored using a Bioscreen growth analyzer with 200 μl of MH broth in 100 well Bioscreen plates inoculated with 2 μl of *B*. *subtilis* strains pre-grown in LB broth at 37°C to an OD_600_ of ~0.4. When required, glucose, MgSO_4_ and IPTG were added to the final concentrations of 1%, 20 mM and 1 mM respectively.

### qRT-PCR

Strains of interest were grown to an OD_600_ of ~0.5. 1.5 ml of culture was used for RNA extraction. RNA isolation (Qiagen, USA) and cDNA preparation (Thermofisher, USA) was carried out as suggested by the manufacturer. qRT-PCR was carried out using a Bio-Rad iTaq universal SYBR green super mix. 23S rRNA was used to normalize the cycle threshold (Ct) value.

### Cell lysate preparation and western blot

For GlmS measurements, Δ*glmR* and Δ*glmR glmS1* strains were grown in LB medium to an OD_600_ of ~0.3 at 37°C with shaking. 30 ml of culture was withdrawn and centrifuged at 5000 rpm for 10 minutes. Cell pellets were frozen at -20°C. Pellets were washed once with 1X phosphate buffer saline (pH 7.4). 150 μl of lysis buffer (20 mM tris-HCl, 100 mM NaCl, 1 mM EDTA, 1 mM DTT, 10% glycerol and protease inhibitor cocktail) was used to re-suspend the cell pellets. One tablet of protease inhibitor cocktail from Roche diagnostics was added to 10 ml of lysis buffer. Cells were lysed by sonication. After centrifugation cell lysates were transferred to fresh tubes. Protein concentration was measured by Bradford assay (Bio-Rad). 5 μg of protein was run on a 4–15% gradient gel from Bio-Rad. Protein was transferred onto a PVDF membrane using a Bio-Rad transblot turbo transfer system. The membrane was blocked with 5% milk powder for one hour followed by overnight incubation with primary anti-GlmS polyclonal antibodies [66] added to 1:3000 dilution in 1X tris buffer saline with 0.1% tween 20 and 0.5% milk powder. After three washes, the membrane was incubated with a 1:3000 dilution of HRP conjugated anti-Rabbit antibodies (Sigma). Bands were visualized on a Bio-Rad Chemidoc MP imaging system.

### GlcNAc disc diffusion assay

Strains of interest were grown in 5 ml LB medium to an OD_600_ of ~0.4. 100 μl of cells were added to 4 ml top MH agar (0.7% agar) preheated at 50°C and was laid on a 15 ml MH agar (1.5%) plate. A disc with 0.5 mg GlcNAc (Sigma, USA) was put on the plate. After overnight incubation at 37°C, the zone of growth surrounding the disc was measured.

### CRISPR editing

DNA changed encoding single amino acid substitutions (GlmR_Y255A,_ GlmR_R301A_ and GlmR_R301E_) were generated at the native *glmR* locus using CRISPR editing as described [67]. In brief, oligonucleotides encoding a 20 nucleotide gRNA with flanking BsaI sites and a repair fragment carrying mutations of interest with flanking SfiI restrictions sites were cloned sequentially into vector pJOE8999 followed by transformation into *E*. *coli* DH5α cells. The resultant plasmid was transformed into recipient *B*. *subtilis* strain and cells were plated on 15 μg/ml kanamycin plates with 0.2% mannose. Transformation was carried out at 30°C as pJOE8999 cannot replicate at higher temperatures. The transformants were patched on LB agar plates and incubated at the non-permissive temperature of 42°C. The loss of vector was confirmed by the inability of selected isolates on kanamycin plates. The presence of the desired mutations was confirmed by Sanger sequencing.

### Bacterial two hybrid

Vectors pT18 and pT25 and strains for bacterial two hybrid were prepared as described [60]. *E*. *coli* BTH101 strains carrying pT18 and pT25 with genes of interest were grown in LB broth overnight at 30°C with 100 μg/ml ampicillin, 50 μg/ml chloramphenicol and 0.5 mM IPTG. 10 μl of cells were spotted on LB plate with 100 μg/ml ampicillin, 50 μg/ml chloramphenicol, 0.5 mM IPTG and 40 μg/ml X-gal. Plates were incubated overnight at 30°C.

### RNA structure analysis

*In silico* analysis was carried out using NUPACK web application [68].

## Supporting information

S1 Table Secondary suppressors of CEF resistance. (DOCX)

S2 Table *B*. *subtilis* strains used in this study. (DOCX)

S1 Fig Chemical suppression of the Δ*glmR* inability to grow on MH medium. (A) Growth curves showing the effect of addition of glucose and MgSO_4_ on growth of Δ*glmR* in MH medium compared to WT. (B) Growth stimulation on MH medium by glucose. Top MH agar (4 ml) was plated with 100 μl of Δ*glmR* cells and filter discs containing 2.5 mg and 5 mg of glucose were put on the plate followed by overnight incubation at 37°C.
(PDF)

S2 Fig Δ*glmR* susceptibility to PG biosynthesis inhibiting antibiotics. Disc diffusion assay for WT and Δ*glmR* done with **(A)** oxacillin (1 μg), **(B)** cefixime (40 μg), **(C)** moenomycin (10 μg), **(D)** vancomycin (10 μg), **(E)** fosfomycin (50 μg), **(F)** bacitracin (400 μg) and **(G)** nisin (50 μg). One asterisk and three asterisks represent significance with P <0.05 and P <0.001 respectively. NS indicates that differences were not significant.
(PDF)

S3 Fig Suppression of Δ*glmR* CEF sensitivity by complementation and by Mg^+2^. **(A)** CEF susceptibility of Δ*glmR* and complementation of phenotype by IPTG inducible ectopic expression of 3X-FLAG *glmR*. RBS was optimized to AGGAGG that is seven base pair apart from start codon. **(B)** Disc diffusion assay showing suppression of CEF sensitivity of *ΔglmR* by addition of 20 mM MgSO_4_. In both figures, statistical significance is indicated by asterisks with P <0.001.
(PDF)

S4 Fig *rho* deletion suppresses *ΔglmR* cefuroxime sensitivity phenotype only if a primary suppressor mutation (*glmS1*) is present. CEF disc diffusion assay showing effect of *rho* mutation. Statistical significance with P <0.001 is indicated with three asterisks.
(PDF)

S5 Fig Point mutations *rsiW1* and *rsiW2* destabilize the *sigW-rsiW* transcription termination loop. **(A)** Transcription termination loop secondary structure prediction for WT and with point mutations (*rsiW1* and *rsiW2*) are shown with their relative free energy value prediction. **(B)** CEF disc diffusion assays performed on strains with *sigW*::*erm* mutation in WT, Δ*glmR*, Δ*glmR rsiW1* and Δ*glmR rsiW2* backgrounds.
(PDF)

S6 Fig Effects of c-di-AMP hydrolase deletion mutations on CEF sensitivity and growth on MH medium for the Δ*glmR* strain. **(A)** CEF susceptibility and **(B)** growth on MH medium for Δ*glmR* in combination with *gdpP* and *pgpH* deletions and the *gdpP pgpH* double deletion.
(PDF)
