# Supplementary material for: A metabolic checkpoint protein GlmR is important for diverting carbon into peptidoglycan biosynthesis in Bacillus subtilis
Source: PLoS Genet. 2018 Sep 24;14(9):e1007689. doi: 10.1371/journal.pgen.1007689 (PMC6171935; doi:10.1371/journal.pgen.1007689)
Supplement: S1 Table — (DOCX) [file pgen.1007689.s001.docx]

**Table S1. Secondary suppressors of CEF resistance**

| Primary CEF^R^ suppressor of Δ*glmR* | ZOI (mm) of Δ*glmR* with primary suppressor | Secondary mutation | Coding region change | ZOI(mm) with primary and secondary suppressors |
| --- | --- | --- | --- | --- |
| *glmS1* | *30* | *rho*_E36fS_ | 105delA | 16 |
| *rsiW1* | *27* | *rho*_R282I_ | 845G>T | 16 |
| *rsiW2* | *27* | *rho*_T337K_ | 1010C>A | 17 |
| *pgcA_G47S_* | *29* | *rho_I_*_288fs_ | 861_862insG | 17 |
| *pgcA_G47S_* | *29* | *rho*_E59K_ | 175G>A | 17 |
