# Supplementary material for: A metabolic checkpoint protein GlmR is important for diverting carbon into peptidoglycan biosynthesis in Bacillus subtilis
Source: PLoS Genet. 2018 Sep 24;14(9):e1007689. doi: 10.1371/journal.pgen.1007689 (PMC6171935; doi:10.1371/journal.pgen.1007689)
Supplement: S2 Table — (DOCX) [file pgen.1007689.s002.docx]

**Table S2. *B. subtilis* strains used in this study**

| Strain | Genotype | Source/ ref |
| --- | --- | --- |
| 168 | *trpC2* | Lab stock |
| HB16822 | *trpC2 rho::erm* | This work |
| HB16825 | *trpC2 glmR rho::erm* | This work |
| HB16848 | *trpC2* *glmR* | This work |
| HB16849 | *trpC2 sigW* | This work |
| HB16850 | *trpC2 glmR* *sigW* | This work |
| HB16858 | *trpC2* 200068A>T (*glmS1*) | This work |
| HB16868 | *trpC2 glmR* *glmS1* | This work |
| HB16905 | *trpC2 glmR* *amyE*::P_spac(hy)_-*cdaA-cdaR* | This work |
| HB16910 | *trpC2 glmR* *amyE*::P_spac(hy)_-*glmM* | This work |
| HB16913 | *trpC2 glmR* *amyE*::P_spac(hy)_-*cdaA* | This work |
| HB16942 | *trpC2* 196071C>T (*rsiW2*) | This work |
| HB16943 | *trpC2 glmR glmS1 rho::erm* | This work |
| HB16950 | *trpC2* *glmR* *rsiW2* | This work |
| HB16951 | *trpC2 glmR* *amyE*::P_spac(hy)_ -*glmR* | This work |
| HB16954 | *trpC2 glmR* *amyE*::P_spac(hy)_-*glmR*_T304A_ | This work |
| HB16955 | *trpC2 glmR* *amyE*::P_spac(hy)_-*glmR*_T304E_ | This work |
| HB16960 | *trpC2* 196049G>A (*rsiW1*) | This work |
| HB16963 | *trpC2 glmR* *amyE*::P_spac(hy)_-*cdaA-cdaR-glmM* | This work |
| HB16964 | *trpC2* *glmR* *amyE*::P_spac(hy)_-*murAA* | This work |
| HB16965 | *trpC2 glmR* *rsiW1* | This work |
| HB16979 | *trpC2 glmR glmS1 amyE::P_spac(hy)_ murAA* | This work |
| HB16994 | *trpC2 glmR* *nagB* | This work |
| HB20901 | *trpC2 nagA* | This work |
| HB20902 | *trpC2 glmR* *nagA* | This work |
| HB20917 | *trpC2 glmR* *nagB* *gamA* | This work |
| HB21902 | *trpC glmR zwf* | This work |
| HB21906 | *trpC2 glmR amyE::P_spac(hy)_ zwf* | This work |
| HB21915 | *trpC2 glmR* *sigW::erm* *rsiW1* | This work |
| HB21916 | *trpC2 glmR* *sigW* *rsiW2* | This work |
| HB21920 | *trpC2 glmR* *sigW* *rsiW1* | This work |
| HB21921 | *trpC2 glmR* *sigW* *rsiW2* | This work |
| HB21922 | *trpC2 glmR* *amyE*::P_spac(hy)_-*glmU* | This work |
| HB21923 | *trpC2 glmR* *amyE*::P_spac(hy)_-*murB* | This work |
| HB21926 | *trpC2 glmR* *amyE*::P_spac(hy)_-*cdaA-cdaR-glmM-glmS* | This work |
| HB21929 | *trpC2 glmR* *glmS1* *amyE*::P_spac(hy)_-*murB* | This work |
| HB21932 | *trpC2 glmR_Y265A_* | This work |
| HB21942 | *trpC glmR* *amyE*::P_spac(hy)_-*glmS* | This work |
| HB21947 | *trpC2 nagB gamA* | This work |
| HB21957 | *trpC2 glmR_R301A_* | This work |
| HB21958 | *trpC2 glmR_R301E_* | This work |
