# Supplementary material for: A metabolic checkpoint protein GlmR is important for diverting carbon into peptidoglycan biosynthesis in Bacillus subtilis
Source: PLoS Genet. 2018 Sep 24;14(9):e1007689. doi: 10.1371/journal.pgen.1007689 (PMC6171935; doi:10.1371/journal.pgen.1007689)
Supplement: S1 Fig — (A) Growth curves showing the effect of addition of glucose and MgSO4 on growth of ΔglmR in MH medium compared to WT. (B) Growth stimulation on MH medium by glucose. Top MH agar (4 ml) was plated with 100 μl of ΔglmR cells and filter discs containing 2.5 mg and 5 mg of glucose were put on the plate followed by overnight incubation at 37°C. (PDF) [file pgen.1007689.s003.pdf]

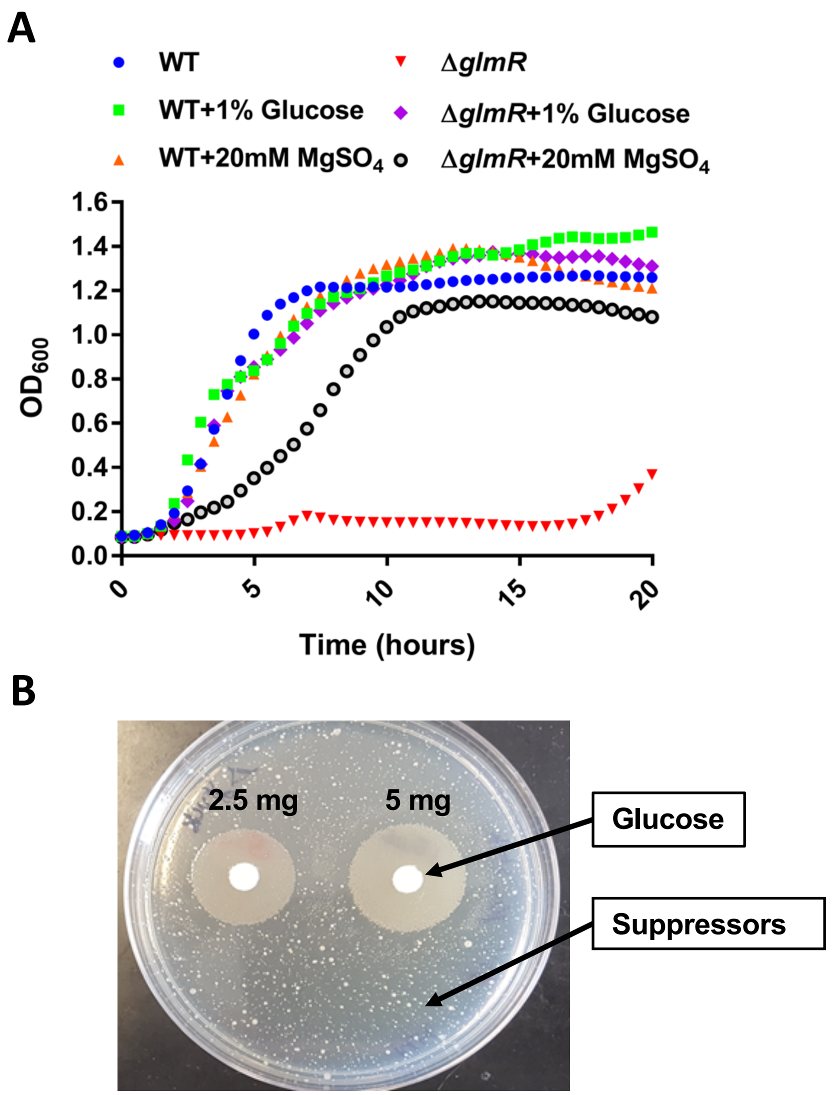

Figure S1: Δ*glmR* is unable to grow on MH medium. (A) Growth curves showing the effect of addition of glucose and $MgSO_4$ on growth of Δ*glmR* in MH medium compared to WT. **(B)** Growth stimulation on MH medium by glucose. Top MH agar (4 ml) was plated with 100 μl of Δ*glmR* cells and filter discs containing 2.5 mg and 5 mg of glucose were put on the plate followed by overnight incubation at 37 ^{0}C.
