# Supplementary material for: A metabolic checkpoint protein GlmR is important for diverting carbon into peptidoglycan biosynthesis in Bacillus subtilis
Source: PLoS Genet. 2018 Sep 24;14(9):e1007689. doi: 10.1371/journal.pgen.1007689 (PMC6171935; doi:10.1371/journal.pgen.1007689)
Supplement: S2 Fig — Disc diffusion assay for WT and ΔglmR done with (A) oxacillin (1 μg), (B) cefixime (40 μg), (C) moenomycin (10 μg), (D) vancomycin (10 μg), (E) fosfomycin (50 μg), (F) bacitracin (400 μg) and (G) nisin (50 μg). One asterisk and three asterisks represent significance with P <0.05 and P <0.001 respectively. NS indicates that differences were not significant. (PDF) [file pgen.1007689.s004.pdf]

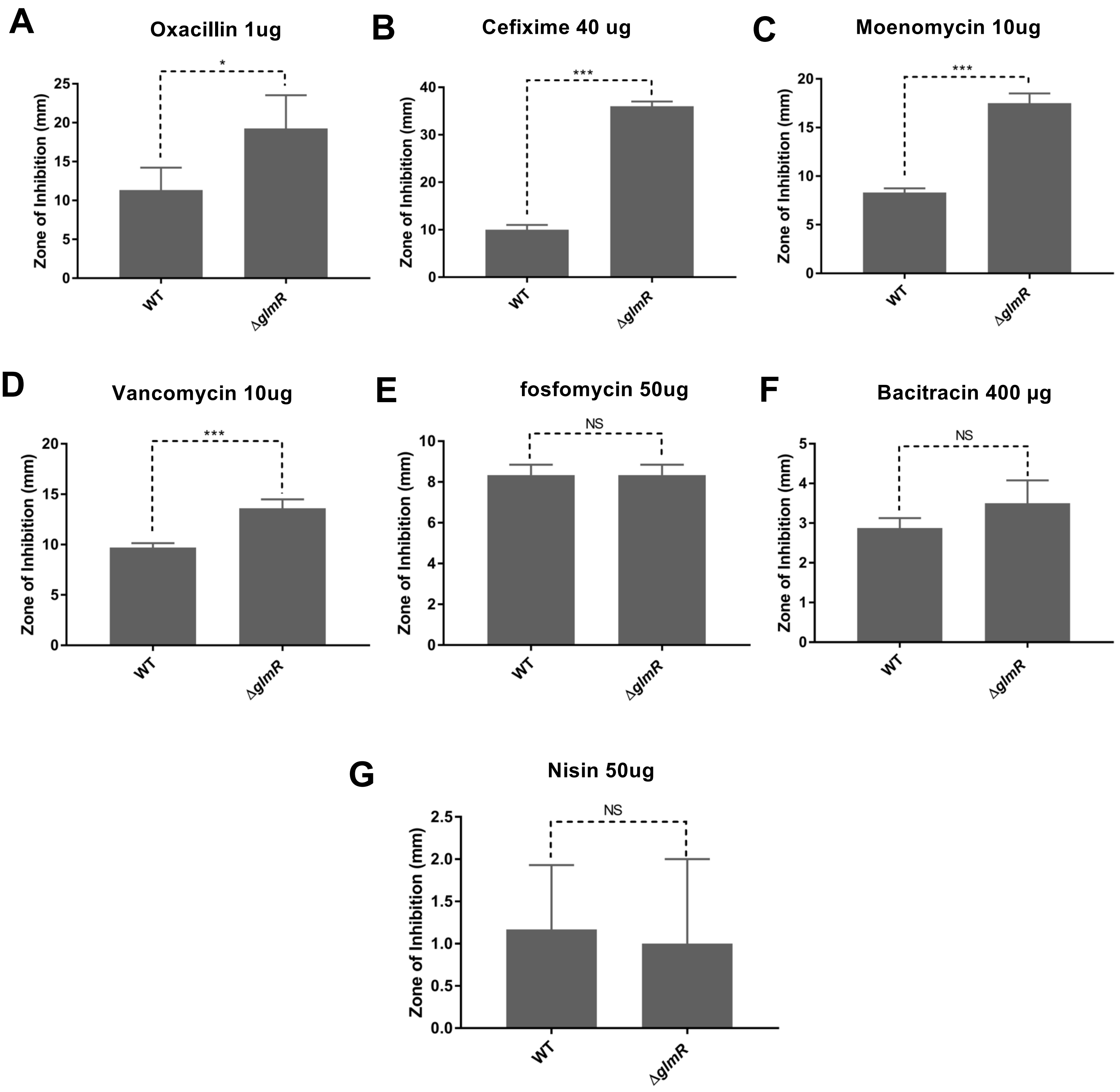

Figure S2: Δ*glmR* susceptibility to PG biosynthesis inhibiting antibiotics. Disc diffusion assay for WT and Δ*glmR* done with **(A)** oxacillin (1 μg), **(B)** cefixime (40 μg), **(C)** moenomycin (10 μg), **(D)** vancomycin (10 μg), **(E)** fosfomycin (50 μg), **(F)** bacitracin (400 μg) and **(G)** nisin (50 μg). One asterisk and three asterisks represent significance with P <0.05 and P <0.001 respectively. NS indicates that differences were not significant.
