# Supplementary material for: A metabolic checkpoint protein GlmR is important for diverting carbon into peptidoglycan biosynthesis in Bacillus subtilis
Source: PLoS Genet. 2018 Sep 24;14(9):e1007689. doi: 10.1371/journal.pgen.1007689 (PMC6171935; doi:10.1371/journal.pgen.1007689)
Supplement: S3 Fig — (A) CEF susceptibility of ΔglmR and complementation of phenotype by IPTG inducible ectopic expression of 3X-FLAG glmR. RBS was optimized to AGGAGG that is seven base pair apart from start codon. (B) Disc diffusion assay showing suppression of CEF sensitivity of ΔglmR by addition of 20 mM MgSO4. In both figures, statistical significance is indicated by asterisks with P <0.001. (PDF) [file pgen.1007689.s005.pdf]

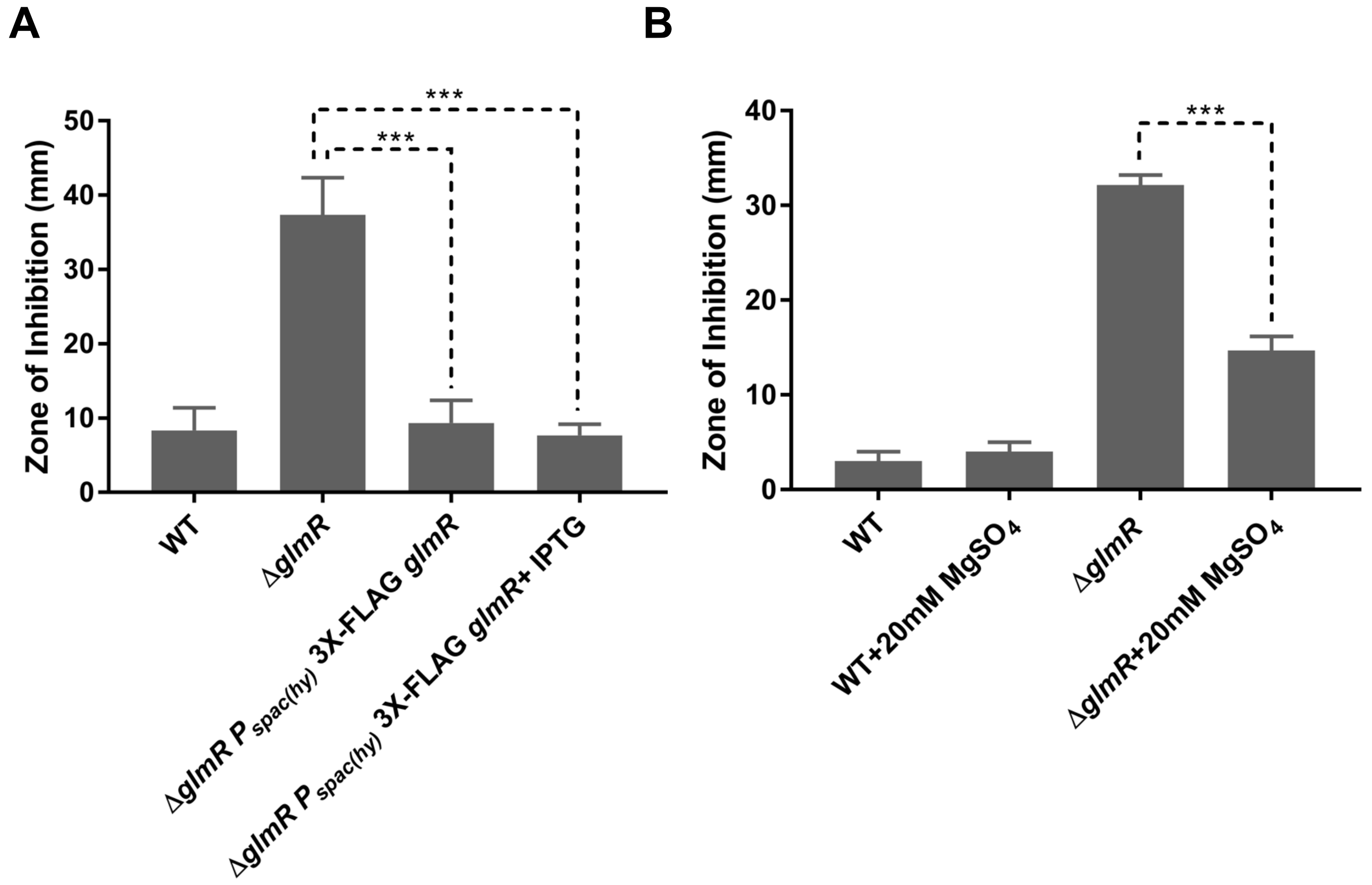

Figure S3: Suppression of Δ*glmR* CEF sensitivity by complementation and by Mg^{+2} (A) CEF susceptibility of Δ*glmR* and complementation of phenotype by IPTG inducible ectopic expression of 3X-FLAG *glmR*. RBS was optimized to AGGAGG that is seven base pair apart from start codon. **(B)** Disc diffusion assay showing suppression of CEF sensitivity of *ΔglmR* by addition of 20 mM $MgSO_4$. In both figures, statistical significance is indicated by asterisks with $P < 0.001$.
