# Supplementary material for: A metabolic checkpoint protein GlmR is important for diverting carbon into peptidoglycan biosynthesis in Bacillus subtilis
Source: PLoS Genet. 2018 Sep 24;14(9):e1007689. doi: 10.1371/journal.pgen.1007689 (PMC6171935; doi:10.1371/journal.pgen.1007689)
Supplement: S4 Fig — CEF disc diffusion assay showing effect of rho mutation. Statistical significance with P <0.001 is indicated with three asterisks. (PDF) [file pgen.1007689.s006.pdf]

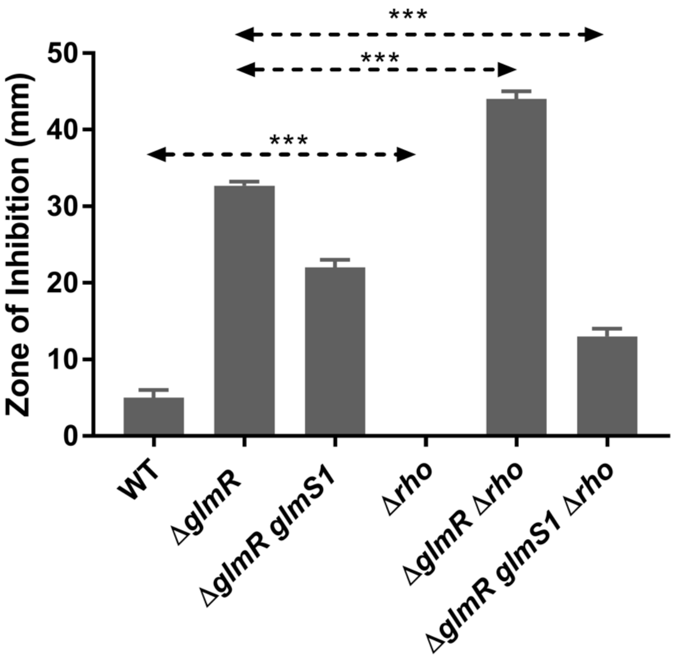

Figure S4: *rho* deletion suppresses *ΔglmR* cefuroxime sensitivity phenotype only if a primary suppressor mutation (*glmS1*) is present. CEF disc diffusion assay showing effect of *rho* mutation. Statistical significance with P <0.001 is indicated with three asterisks.
