# Supplementary material for: A metabolic checkpoint protein GlmR is important for diverting carbon into peptidoglycan biosynthesis in Bacillus subtilis
Source: PLoS Genet. 2018 Sep 24;14(9):e1007689. doi: 10.1371/journal.pgen.1007689 (PMC6171935; doi:10.1371/journal.pgen.1007689)
Supplement: S5 Fig — (A) Transcription termination loop secondary structure prediction for WT and with point mutations (rsiW1 and rsiW2) are shown with their relative free energy value prediction. (B) CEF disc diffusion assays performed on strains with sigW::erm mutation in WT, ΔglmR, ΔglmR rsiW1 and ΔglmR rsiW2 backgrounds. (PDF) [file pgen.1007689.s007.pdf]

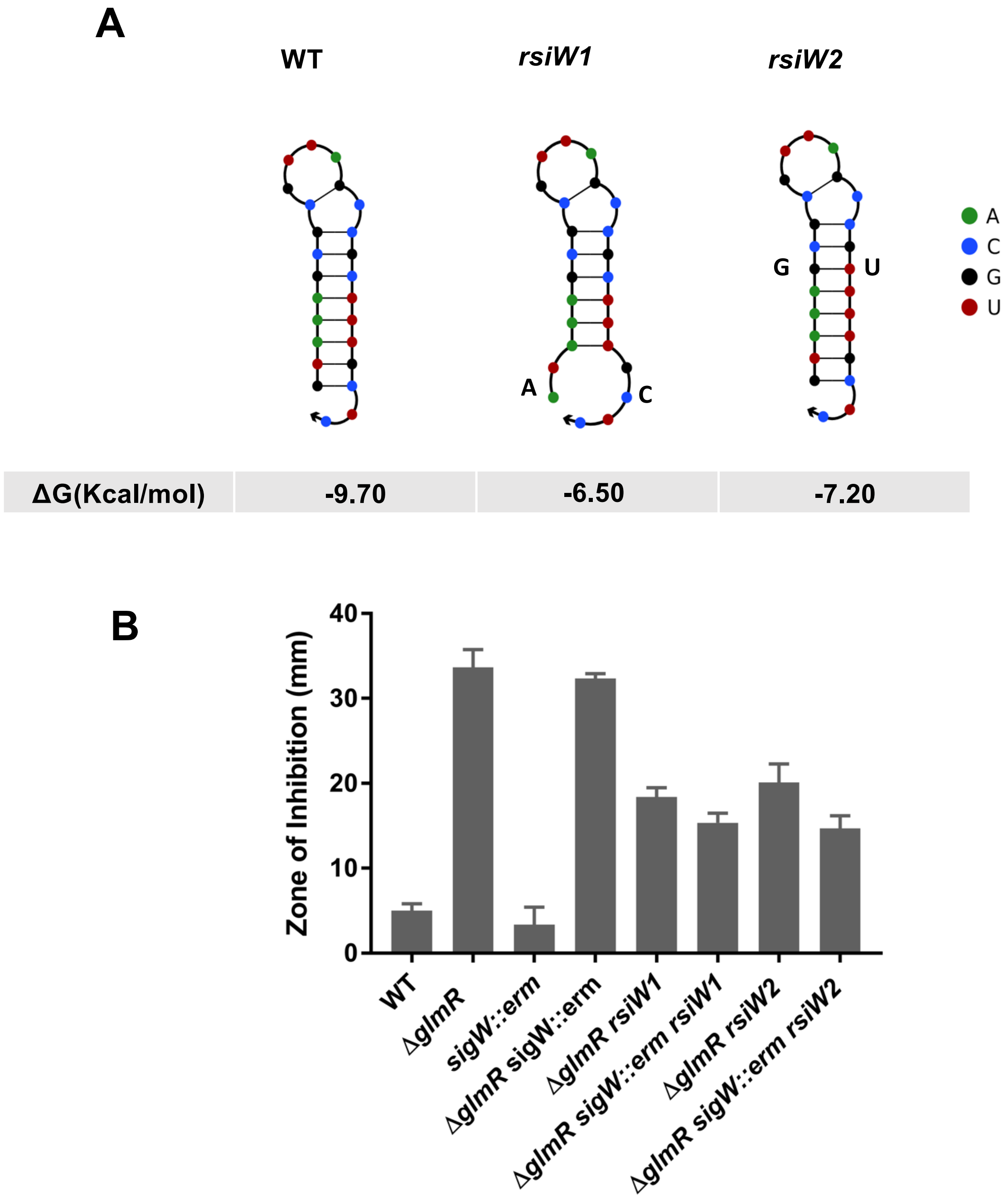

Figure S5: Point mutations *rsiW1* and *rsiW2* destabilize the *sigW-rsiW* transcription termination loop. (A) Transcription termination loop secondary structure prediction for WT and with point mutations (*rsiW1* and *rsiW2*) are shown with their relative free energy value prediction. **(B)** CEF disc diffusion assays performed on strains with *sigW*::*erm* mutation in WT, Δ*glmR*, Δ*glmR rsiW1* and Δ*glmR rsiW2* backgrounds.
