# Supplementary material for: A metabolic checkpoint protein GlmR is important for diverting carbon into peptidoglycan biosynthesis in Bacillus subtilis
Source: PLoS Genet. 2018 Sep 24;14(9):e1007689. doi: 10.1371/journal.pgen.1007689 (PMC6171935; doi:10.1371/journal.pgen.1007689)
Supplement: S6 Fig — (A) CEF susceptibility and (B) growth on MH medium for ΔglmR in combination with gdpP and pgpH deletions and the gdpP pgpH double deletion. (PDF) [file pgen.1007689.s008.pdf]

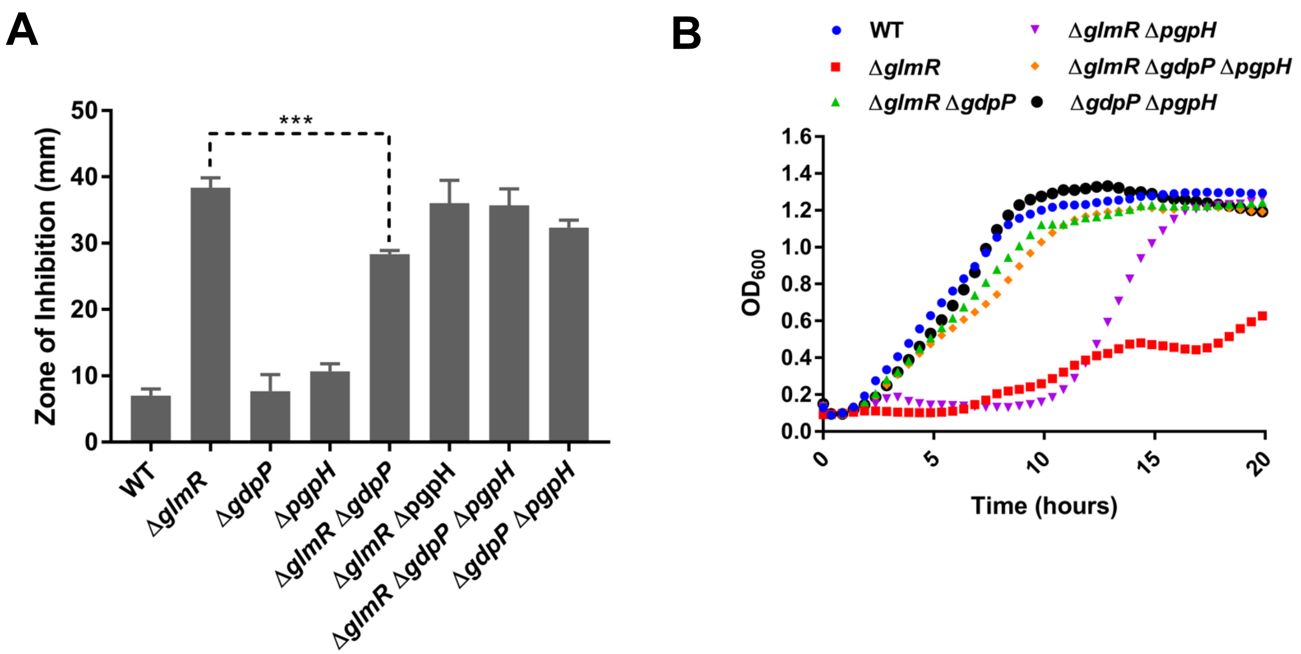

Figure S6: Effects of c-di-AMP hydrolase deletion mutations on CEF sensitivity and growth on MH medium for the Δ*glmR* strain. **(A)** CEF susceptibility and **(B)** growth on MH medium for Δ*glmR* in combination with *gdpP* and *pgpH* deletions and the *gdpP pgpH* double deletion.
